# Supplementary material for: Multi-step control of homologous recombination via Mec1/ATR suppresses chromosomal rearrangements
Source: EMBO J. 2024 Jun 5;43(14):3027–43. doi: 10.1038/s44318-024-00139-9 (PMC11251156; doi:10.1038/s44318-024-00139-9)
Supplement: Supplementary file 1 — Appendix [file 44318_2024_139_MOESM1_ESM.pdf]

Appendix for

Multi-Step Control of Homologous Recombination Via Mec1/ATR Suppresses Chromosomal Rearrangements

Bokun Xie1, Ethan James Sanford1, Shih-Hsun Hung2, Mateusz Maciej Wagner1, Wolf-Dietrich Heyer2 & Marcus B. Smolka1,*

1Department of Molecular Biology and Genetics, Weill Institute for Cell and Molecular Biology, Cornell University, Ithaca, NY, USA
2Department of Microbiology and Molecular Genetics, University of California, Davis, Davis, CA, USA

TABLE OF CONTENTS

Appendix Figure S1 ········· 2
Appendix Figure S2 ········· 4
Appendix Figure S3 ········· 6
Appendix Figure S4 ········· 8
Appendix Figure S5 ········· 10
Appendix Figure S6 ········· 12
Appendix Figure S7 ········· 14
Appendix Figure S8 ········· 16
Appendix Figure S9 ········· 18
Appendix Figure S10 ········· 20
Appendix Figure S11 ········· 22
Appendix Figure S12 ········· 24
Appendix Figure S13 ········· 26
Reference ········· 28

Appendix Figure S1, related to Figure 1

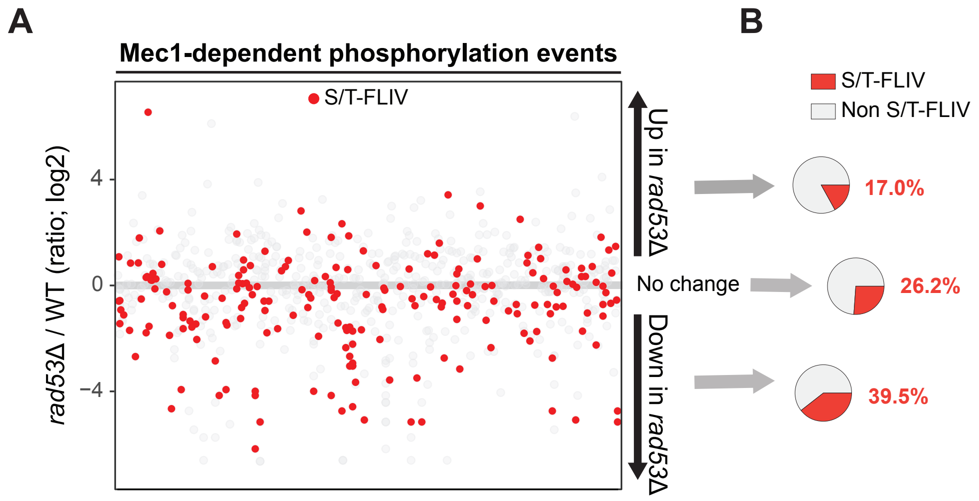

Appendix Figure S1, related to Figure 1. Rad53-dependent signaling enriched for S/T-FLIV motifs is down-regulated in *rad53*Δ cells.

(A) Quantitative phosphoproteomic data showing the modulation of Mec1-dependent phosphorylation events in cells lacking *RAD53*, with S/T-FLIV consensus motifs (preferential Rad53 phosphorylation sites) indicated in red. Cells were treated with MMS for 2 h. The complete list is available in Dataset EV1. (B) Pie chart showing that S/T-FLIV consensus phosphorylation events are downregulated in *rad53*Δ cells.

Appendix Figure S2, related to Figure 1

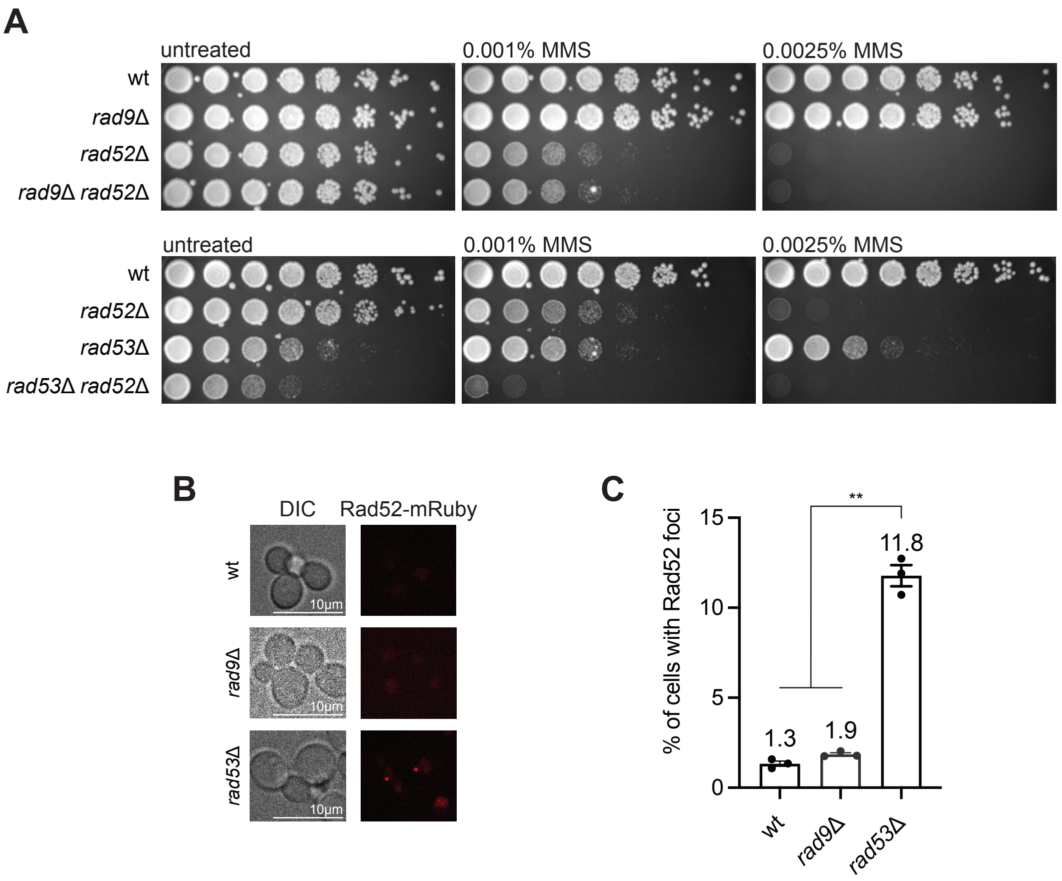

Appendix Figure S2, related to Figure 1. *rad53*Δ cells, but not *rad9*Δ cells, display increased demand for HR.

(A) Dilution assay of cells with indicated genotype in the presence of MMS. (B) Representative image of Rad52 foci in cells with indicated genotype under untreated condition. (C) Quantification of percentages of cells with Rad52 foci. Over 150 cells were scored per replicate. Bars represent mean values and error bars represent standard error of the mean from 3 replicate experiments. P value was calculated using a two-tailed, unpaired t-test. **$P \leq 0.01$.

Appendix Figure S3, related to Figure 1

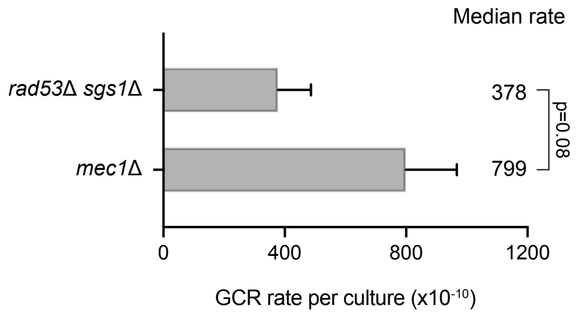

Appendix Figure S3, related to Figure 1. The GCR rate of *rad53*Δ *sgs1*Δ differs from that of *mec1*Δ.

Measurement of GCR rates of *rad53*Δ *sgs1*Δ cells and *mec1*Δ cells. Bars represent median values and error bars represent standard deviation from 32 independent colonies. P value was calculated using a two-tailed, unpaired t-test.

Appendix Figure S4, related to Figure 2

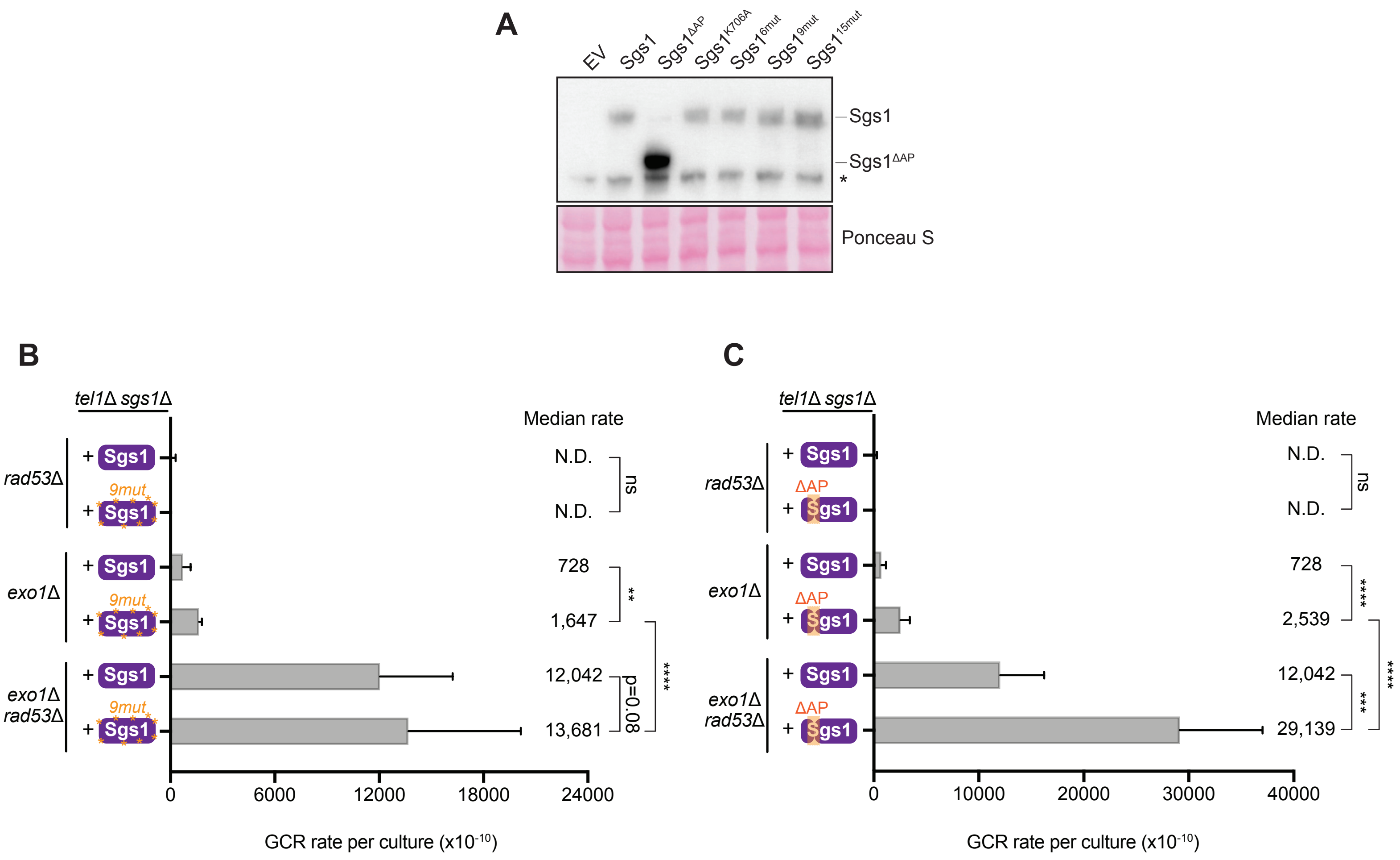

Appendix Figure S4, related to Figure 2. The effect of Sgs1 regulation by Mec1 on GCR suppression in *rad53*Δ cells.

(A) Expression level of different Sgs1 mutants used in this study. Expression of all Sgs1 mutants is driven by the endogenous *SGS1* promoter. (*) Non-specific band detected by the anti-Myc antibody. (B) Measurement of GCR rates in *rad53*Δ cells with the indicated genotypes expressing either Sgs1 or Sgs1^{9mut}. Bars represent median values and error bars represent standard deviation from 32 independent colonies. P value was calculated using a two-tailed, unpaired t-test. **P ≤ 0.01; ****P ≤ 0.0001. (C) Measurement of GCR rates in *rad53*Δ cells with the indicated genotypes expressing either Sgs1 or Sgs1$^{AP\Delta}$. Bars represent median values and error bars represent standard deviation from 32 independent colonies. P value was calculated using a two-tailed, unpaired t-test. ***P ≤ 0.001; ****P ≤ 0.0001.

Appendix Figure S5, related to Figure 2

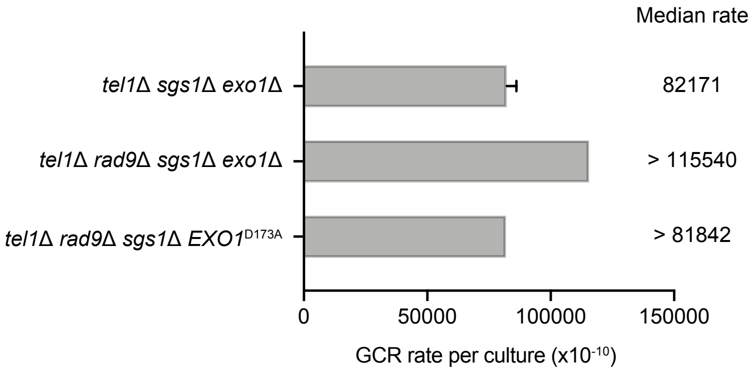

Appendix Figure S5, related to Figure 2. The nuclease activity of Exo1 is important for GCR suppression in the absence of Sgs1.
Measurement of GCR rates of strains with indicated genotype. Bars represent median values and error bars represent standard deviation from 32 independent colonies.

Appendix Figure S6, related to Figure 3

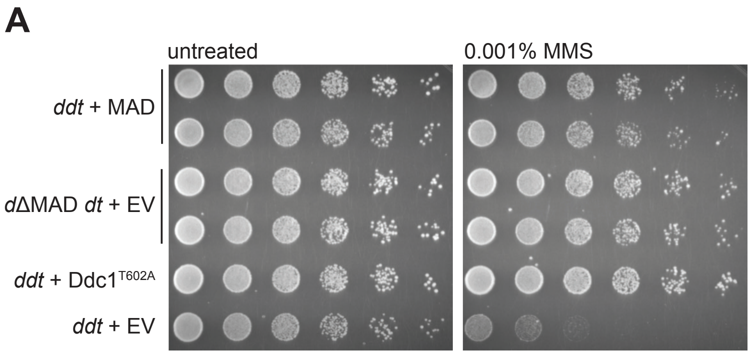

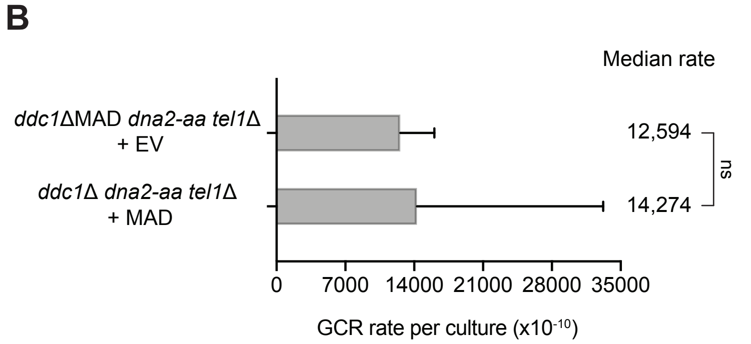

Appendix Figure S6, related to Figure 3. Effects of genomic integration of a Mec1-Activation Domain (MAD) on cell proliferation and GCR rates.

(A) Dilution assay of *ddc1*Δ *dna2-aa tel1*Δ (*ddt*) or *ddc1*ΔMAD *dna2-aa tel1*Δ (*d*ΔMAD *dt*) cells expressing either empty vector, MAD or $Ddc1^{T602A}$ in the presence of MMS. 2-fold serial dilutions were used. (B) Measurement of GCR rates in cells with the indicated genotypes expressing either empty vector or MAD. Bars represent median values and error bars represent standard deviation from 32 independent colonies. P value was calculated using a two-tailed, unpaired t-test. See Lanz *et al*, 2018 for more details on the generation of *ddt* cells and effects of MAD expression.

Appendix Figure S7, related to Figure 3

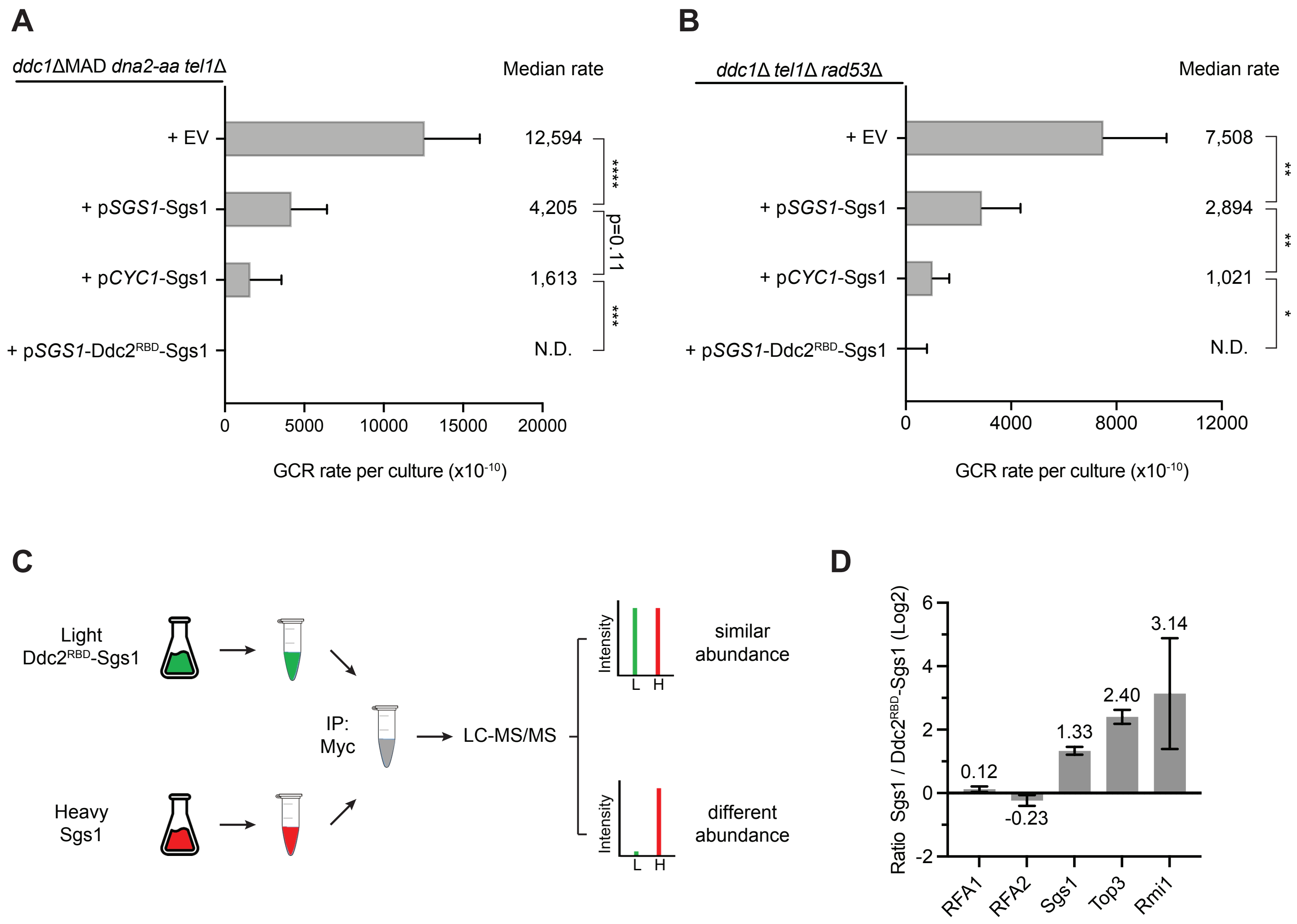

Appendix Figure S7, related to Figure 3. Fusion of RBD to Sgs1 inhibits GCRs independently of abundance changes.

(A) Measurement of GCR rates in *ddc1*ΔMAD *dna2-aa tel1*Δ cells expressing empty vector, p*SGS1::SGS1*, p*CYC1::SGS1* and p*SGS1::RBD-SGS1*. Bars represent median values and error bars represent standard deviation from 32 independent colonies. P value was calculated using a two-tailed, unpaired t-test. ***P ≤ 0.001; ****P ≤ 0.0001. (B) Measurement of GCR rates in *ddc1*Δ *tel1*Δ *rad53*Δ cells expressing empty vector, p*SGS1::SGS1*, p*CYC1::SGS1* and p*SGS1::RBD-SGS1*. Bars represent median values and error bars represent standard deviation from 32 independent colonies. P value was calculated using a two-tailed, unpaired t-test. *P ≤ 0.05; **P ≤ 0.01. (C) Workflow of the SILAC quantitative mass spectrometry method used to measure the abundance of Sgs1 and RBD-Sgs1. (D) Quantitative mass spectrometry analysis of protein abundance from Sgs1-Myc pull-down experiment. Expression of both Sgs1 and RBD-Sgs1 is driven by the endogenous *SGS1* promoter. Error bars represent standard deviation of two or more independent peptide-spectrum matches (PSMs) corresponding to the indicated protein. The expression level of RBD-Sgs1 is about half that of Sgs1.

Appendix Figure S8, related to Figure 3

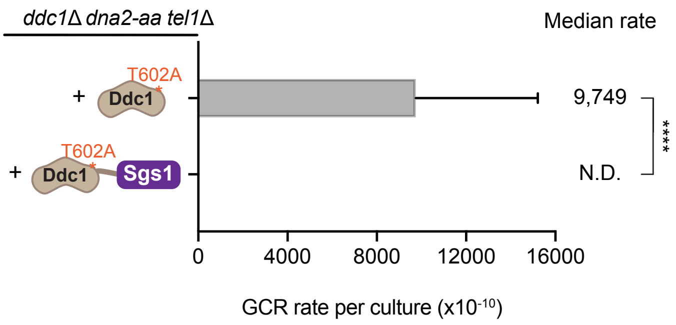

Appendix Figure S8, related to Figure 3. Sgs1 recruitment via fusion with Ddc1 suppresses GCRs in Mec1-deficient cells.

Measurement of GCR rates in *ddc1*Δ *dna2-aa tel1*Δ cells expressing either Ddc1^{T602A} or Ddc1^{T602A}-Sgs1. Bars represent median values and error bars represent standard deviation from 32 independent colonies. P value was calculated using a two-tailed, unpaired t-test. ****P ≤ 0.0001.

Appendix Figure S9, related to Figure 4

A

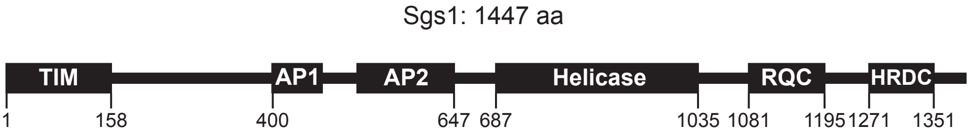

B

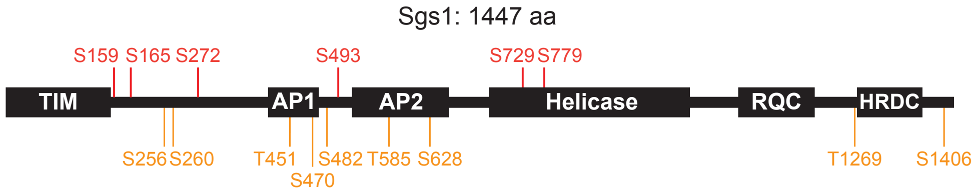

Appendix Figure S9, related to Figure 4. Protein domains and phospho-mutant sites of Sgs1.
(A) Schematics depicting Sgs1 domains. (B) Schematics indicating the position of phosphorylation sites mutated in this study. Orange sites represent all SQ/TQ sites (*9mut*) used in this study. Red sites represent four S-P sites (putative CDK motif) and two other non-SQ/TQ sites detected by mass spectrometry, resulting in the *6mut* Mutant.

Appendix Figure S10, related to Figure 4

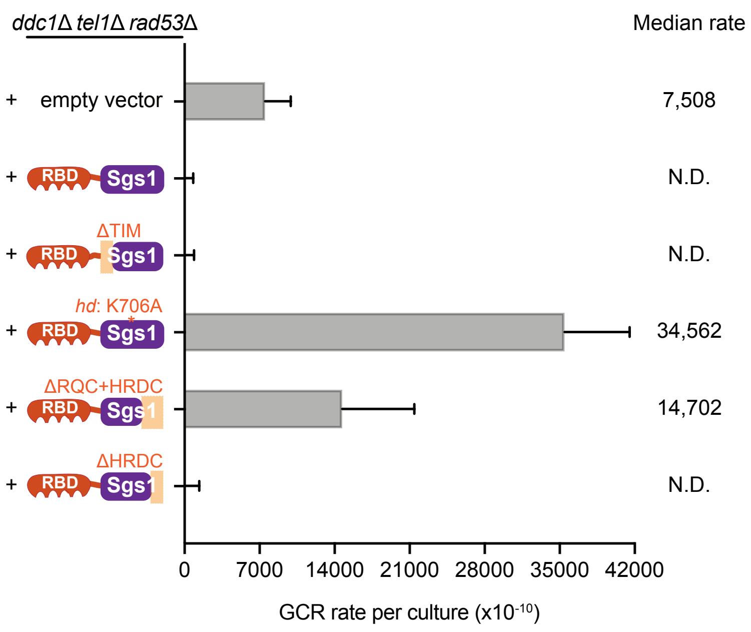

Appendix Figure S10, related to Figure 4. GCR suppression via RBD-Sgs1 requires Sgs1 helicase activity.

Measurement of GCR rates in *ddc1*Δ *tel1*Δ *rad53*Δ cells expressing RBD fused to wild-type or truncations of Sgs1. Bars represent median values and error bars represent standard deviation from 32 independent colonies.

Appendix Figure S11, related to Figure 5

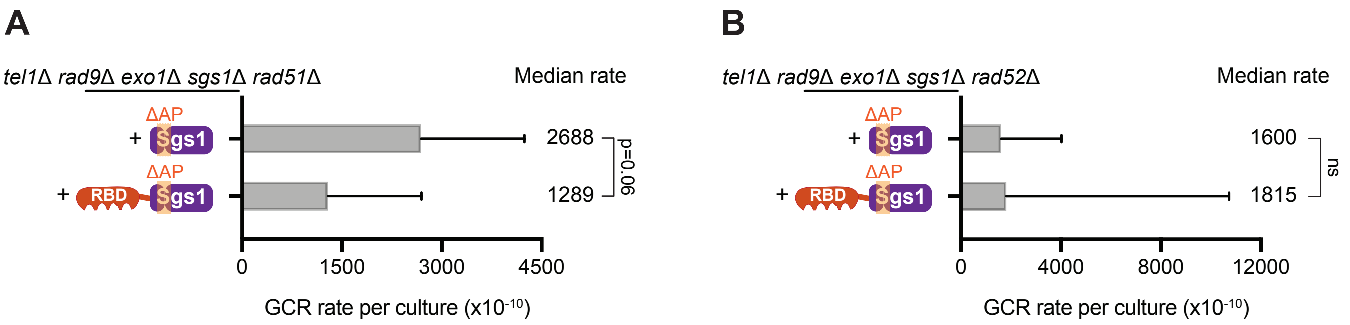

Appendix Figure S11, related to Figure 5. RBD-Sgs1 suppresses HR-driven GCRs.

(A) Measurement of GCR rates in *tel1*Δ *rad9*Δ *exo1*Δ *sgs1*Δ *rad51*Δ cells (cells with deregulated resection) expressing either Sgs1$^{AP\Delta}$ or RBD-Sgs1$^{AP\Delta}$. Bars represent median values and error bars represent standard deviation from 16 independent colonies. P value was calculated using a two-tailed, unpaired t-test. (B) Measurement of GCR rates in *tel1*Δ *rad9*Δ *exo1*Δ *sgs1*Δ *rad52*Δ cells (cells with deregulated resection) expressing either Sgs1$^{AP\Delta}$ or RBD-Sgs1$^{AP\Delta}$. Bars represent median values and error bars represent standard deviation from 16 independent colonies. P value was calculated using a two-tailed, unpaired t-test.

Appendix Figure S12, related to Figure 5

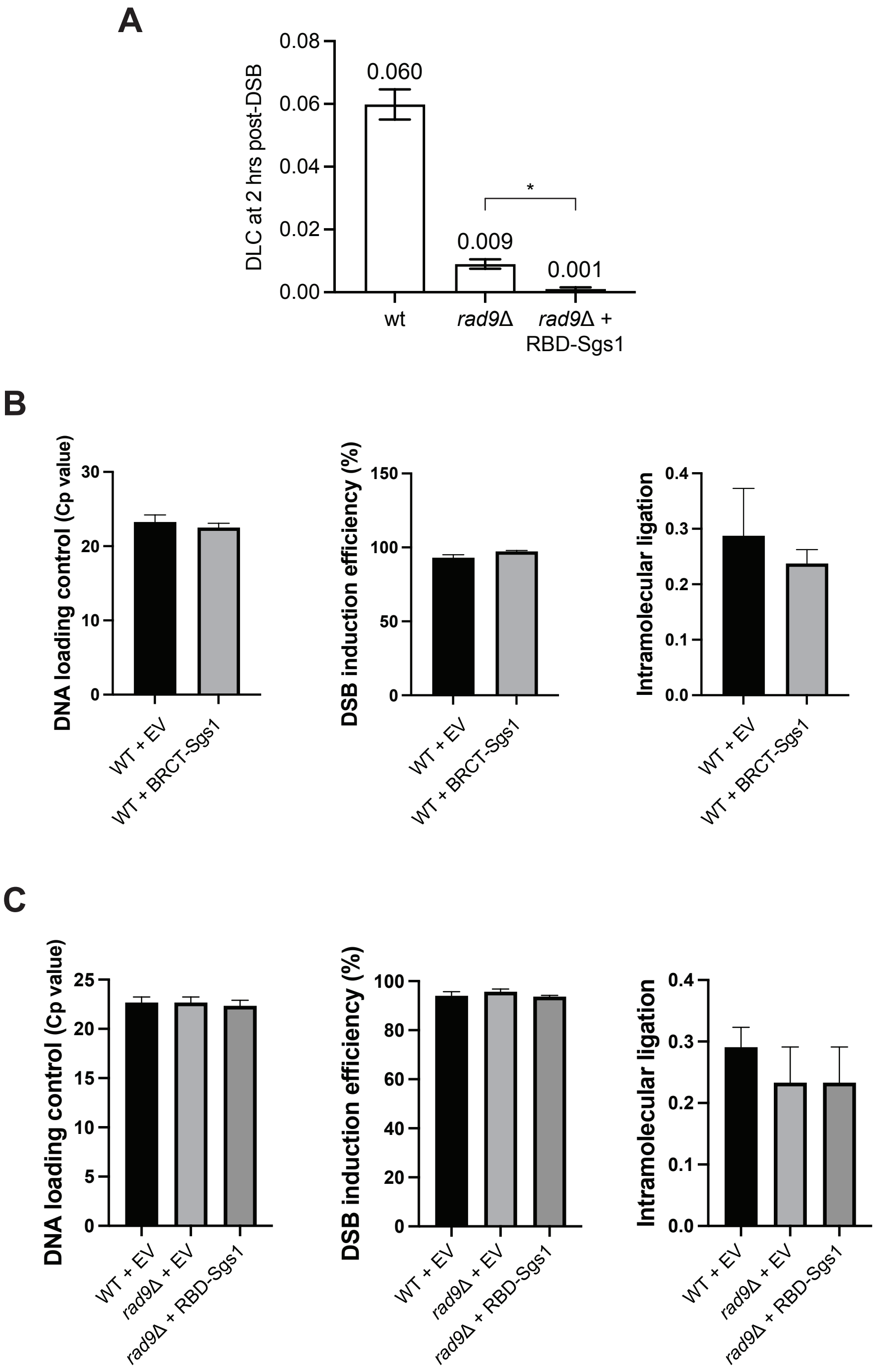

Appendix Figure S12, related to Figure 5. RBD-Sgs1 disrupts D-loop formation.

(A) DLC signal in *rad9*Δ cells carrying an empty vector or expressing the RBD-Sgs1 chimera. Error bars represent SEM of two replicate experiments. P value was calculated with a two-tailed, unpaired t-test. *$P \le 0.05$. (B) Control experiments related to figure 5D. The DNA loading control experiment measures the amplification cycle (Cp value) of the control locus, ARG4. The efficiency of double-strand break (DSB) induction is determined by comparing the percent amplification from the HO cut site at 2 hours to that at 0 hours. Additionally, the intramolecular ligation efficiency assesses the ligation efficiency of a circularized DNA fragment from chromosome VIII following EcoR1 digestion (Piazza *et al*, 2019; Reitz *et al*, 2022). (C) Control experiments related to Appendix Figure S9A. See (B) for details.

Appendix Figure S13, related to Figure 6

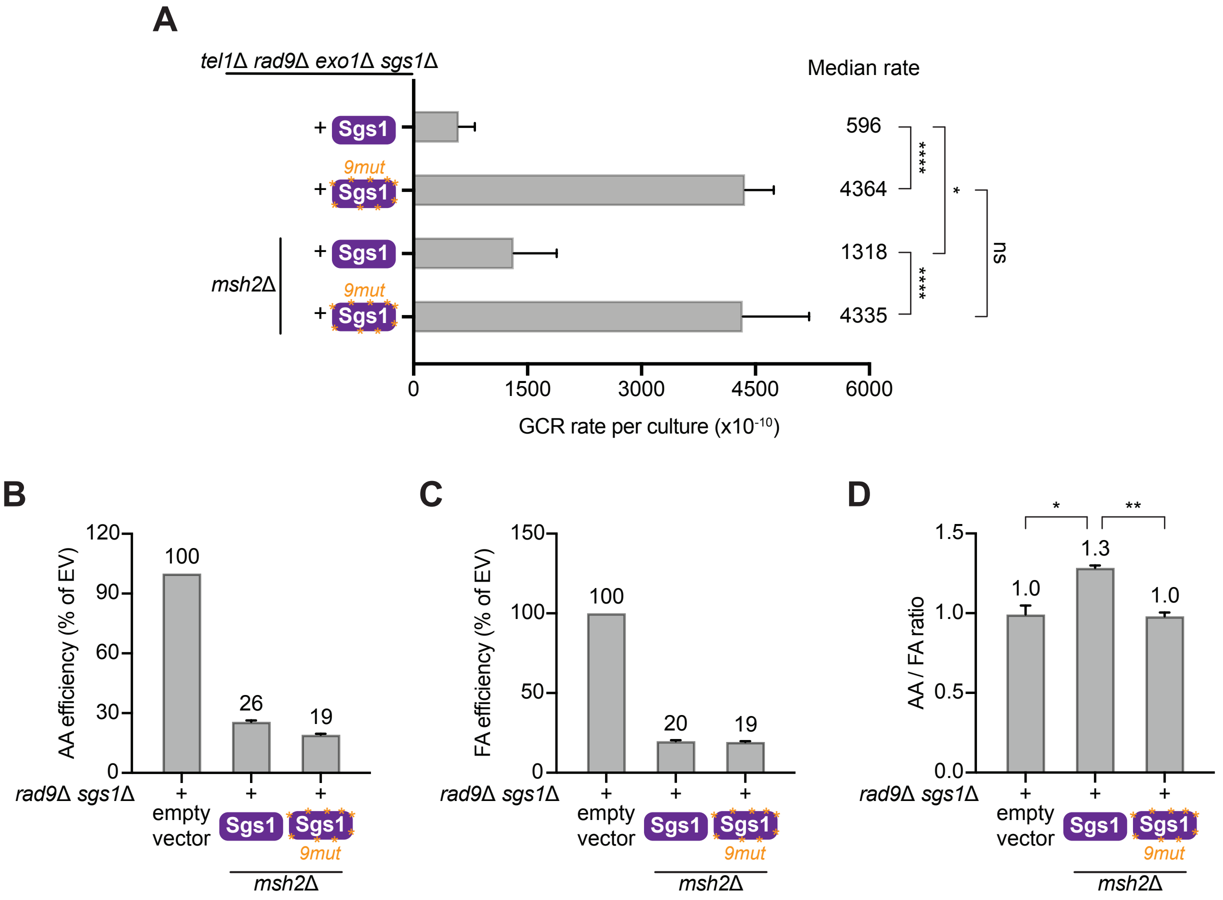

Appendix Figure S13, related to Figure 6. Mec1 phosphorylation of Sgs1 promotes GCR suppression in a manner that is at least partially independent of Msh2.

(A) Measurement of GCR rates in *tel1*Δ *rad9*Δ *exo1*Δ *sgs1*Δ *msh2*Δ cells expressing Sgs1 or Sgs1^{9mut}. Bars represent median values and error bars represent standard deviation from 32 independent colonies. P value was calculated using a two-tailed, unpaired t-test. *P ≤ 0.05; ****P ≤ 0.0001. (B) Measurement of SSA efficiency in the AA strains expressing an empty vector or Sgs1 mutants. Bars represent mean values and error bars represent standard error of the mean from 4 replicate experiments. (C) Measurement of SSA efficiency in the FA strains expressing an empty vector or Sgs1 mutants. Bars represent mean values and error bars represent standard error of the mean from 4 replicate experiments. (D) AA/FA ratio computed from data in (B) & (C). P value was calculated using a two-tailed, unpaired t-test. *P ≤ 0.05; **P ≤ 0.01.

Reference

Lanz MC, Oberly S, Sanford EJ, Sharma S, Chabes A & Smolka MB (2018) Separable roles for Mec1/ATR in genome maintenance, DNA replication, and checkpoint signaling. *Genes Dev* 32: 822–835

Piazza A, Shah SS, Wright WD, Gore SK, Koszul R & Heyer W-D (2019) Dynamic Processing of Displacement Loops during Recombinational DNA Repair. *Mol Cell* 73: 1255–1266.e4

Reitz D, Savocco J, Piazza A & Heyer W-D (2022) Detection of Homologous Recombination Intermediates via Proximity Ligation and Quantitative PCR in Saccharomyces cerevisiae. *J Vis Exp*
