# Supplementary material for: Multi-step control of homologous recombination via Mec1/ATR suppresses chromosomal rearrangements
Source: EMBO J. 2024 Jun 5;43(14):3027–43. doi: 10.1038/s44318-024-00139-9 (PMC11251156; doi:10.1038/s44318-024-00139-9)
Supplement: Supplementary file 8 — Source data Fig. 3 [file 44318_2024_139_MOESM8_ESM.zip › Fig 3 data/Fig 3G_data/Readme_3G.rtf]

For the Rad52 foci quantification, we apply maximum projection. We insert the DIC (bright field) images into the fluorescent images. We use green channel for DIC and red channel for mRuby signal. We need to use overlap to accurately count foci inside the cells. We use green channel for DIC because the images get over-exposed if we overlap the original DIC channel with the red channel.
