# Supplementary material for: Multi-step control of homologous recombination via Mec1/ATR suppresses chromosomal rearrangements
Source: EMBO J. 2024 Jun 5;43(14):3027–43. doi: 10.1038/s44318-024-00139-9 (PMC11251156; doi:10.1038/s44318-024-00139-9)
Supplement: Supplementary file 8 — Source data Fig. 3 [file 44318_2024_139_MOESM8_ESM.zip › Fig 3 data/Figure3H_data/Rad53_activation_rep2/loading order.rtf]

Loading order:wt+EVwt+EV+0.02%MMSwt+Sgs1wt+Sgs1+0.02%MMSwt+RBD-Sgs1wt+RBD-Sgs1+0.02%MMS
