# Supplementary figures and images for: Multi-step control of homologous recombination via Mec1/ATR suppresses chromosomal rearrangements (part 1 of 2)
Source: EMBO J. 2024 Jun 5;43(14):3027–43. doi: 10.1038/s44318-024-00139-9 (PMC11251156; doi:10.1038/s44318-024-00139-9)

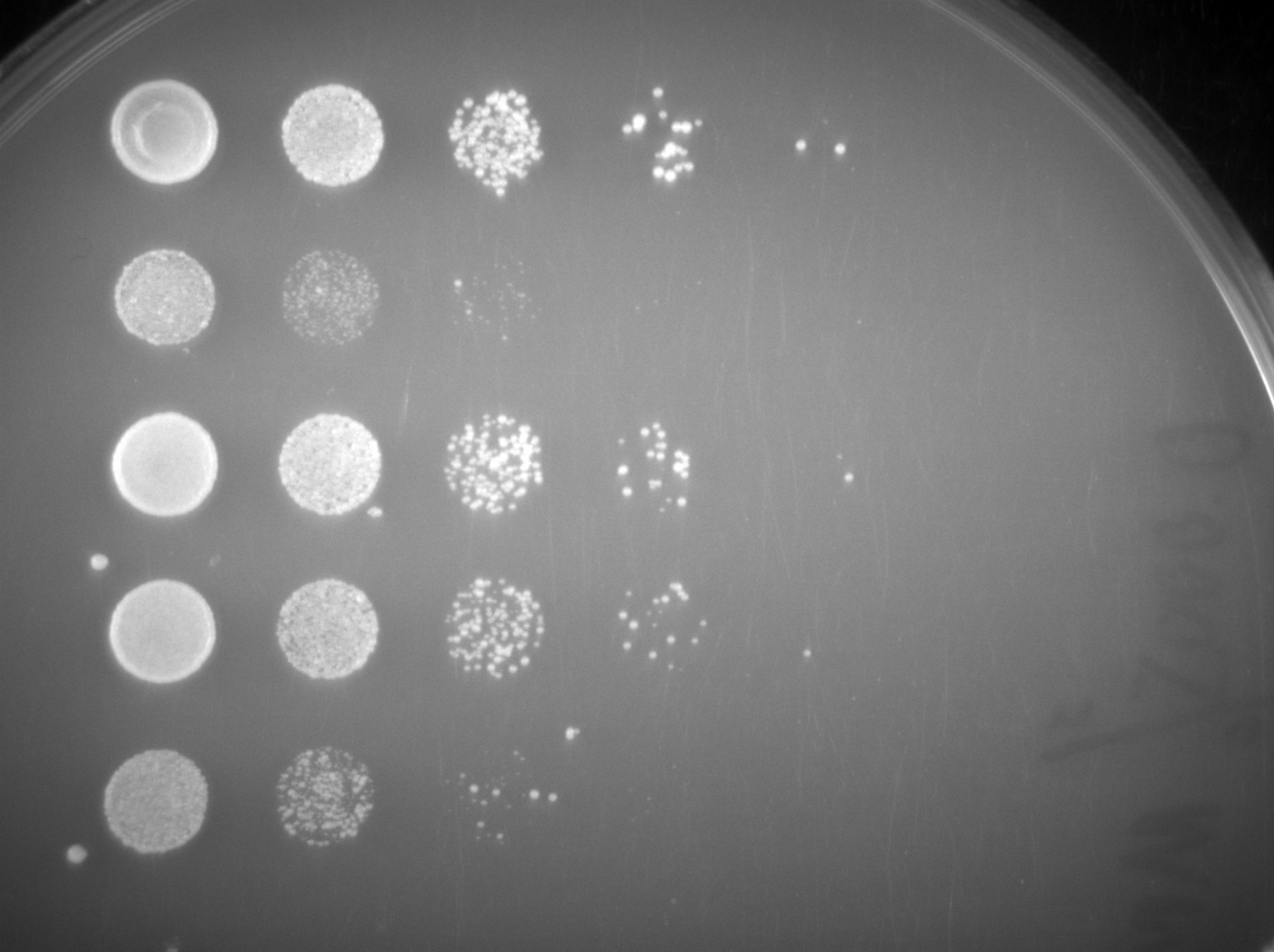

Supplement: Supplementary file 8 — Source data Fig. 3 [file 44318_2024_139_MOESM8_ESM.zip › Fig 3 data/Figure3E_data/Sgs1 rejector sensitivity-mec1/00005mms.jpg]

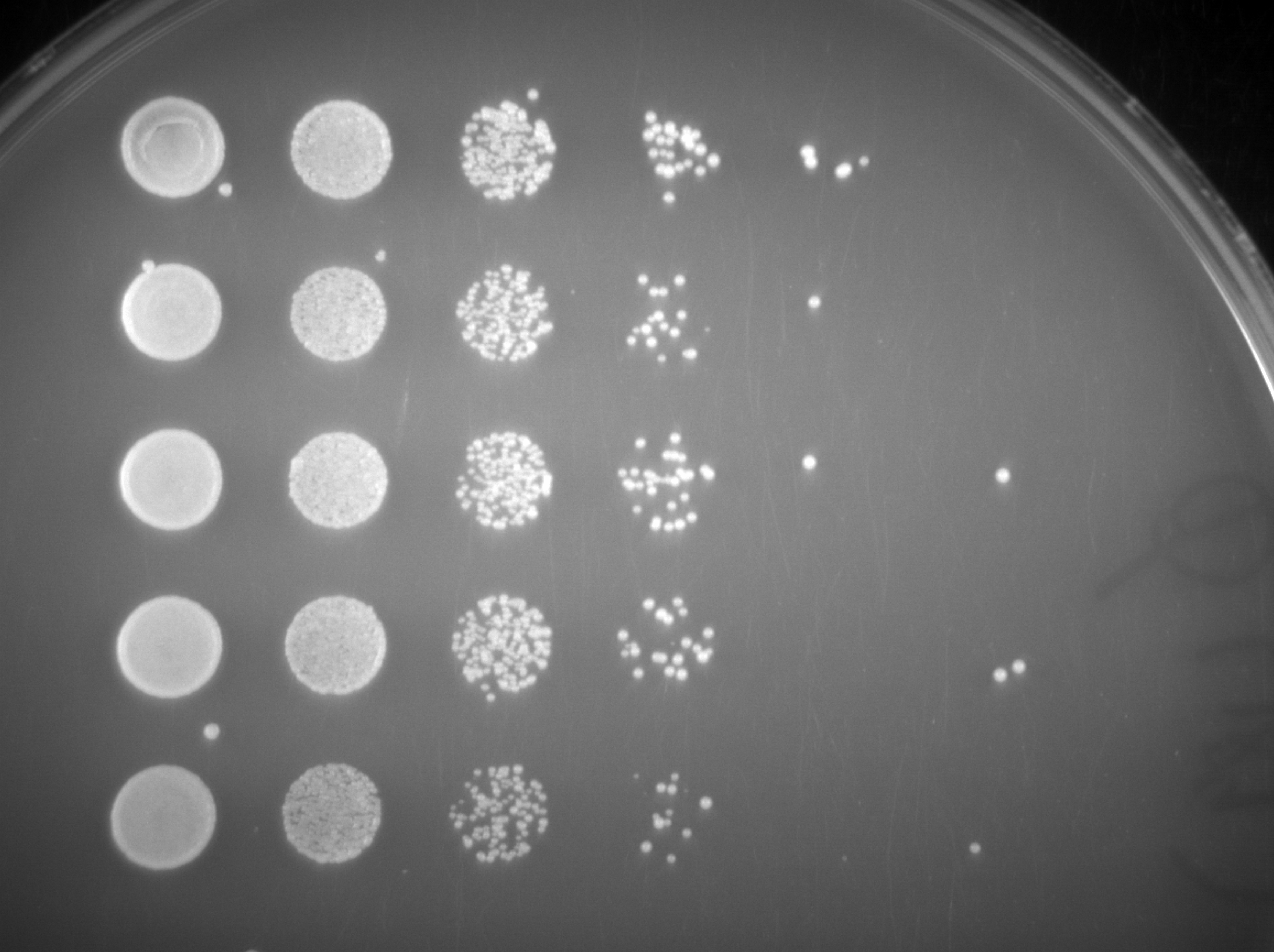

Supplement: Supplementary file 8 — Source data Fig. 3 [file 44318_2024_139_MOESM8_ESM.zip › Fig 3 data/Figure3E_data/Sgs1 rejector sensitivity-mec1/untreated.jpg]

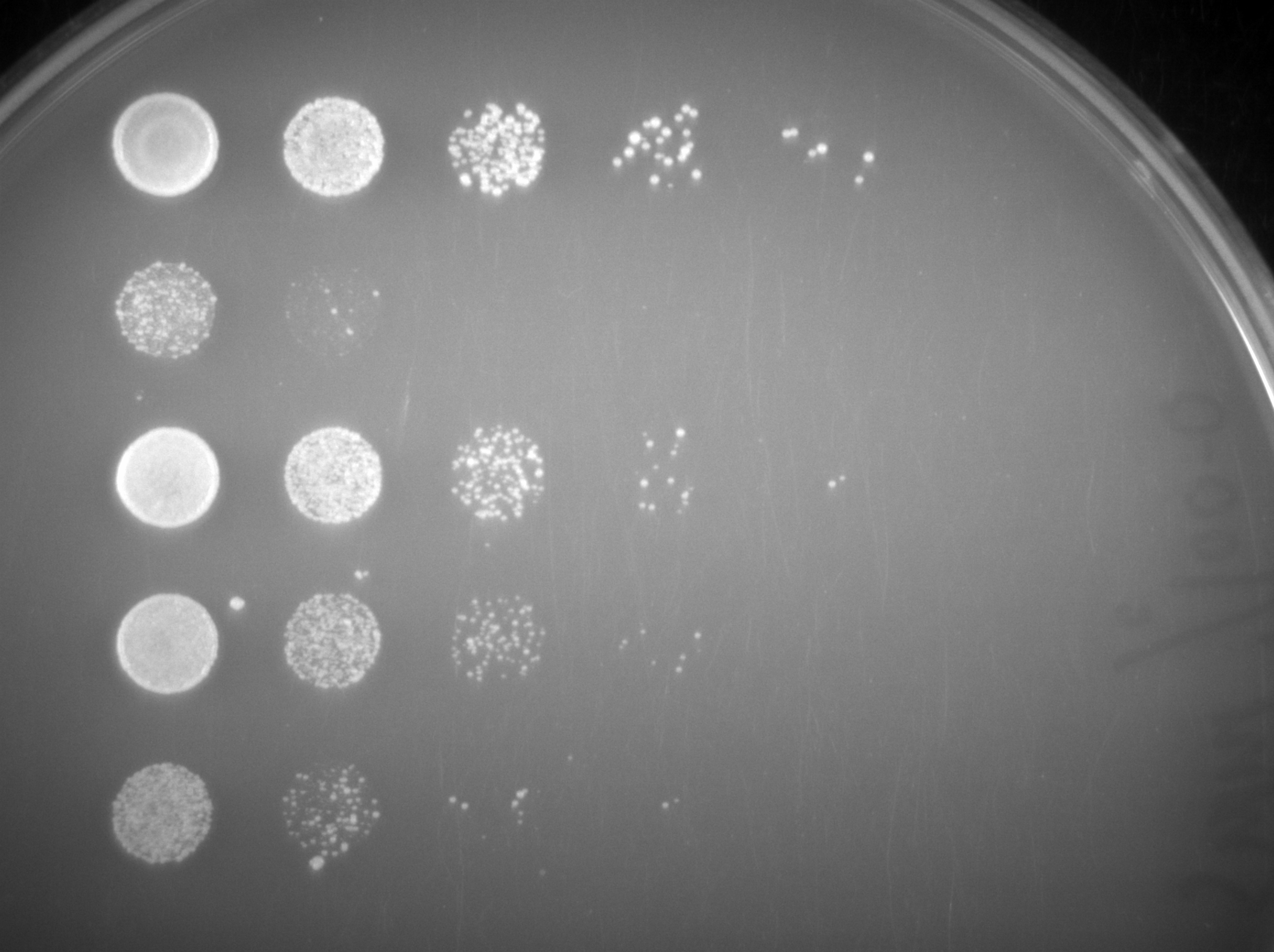

Supplement: Supplementary file 8 — Source data Fig. 3 [file 44318_2024_139_MOESM8_ESM.zip › Fig 3 data/Figure3E_data/Sgs1 rejector sensitivity-mec1/0001mms.jpg]

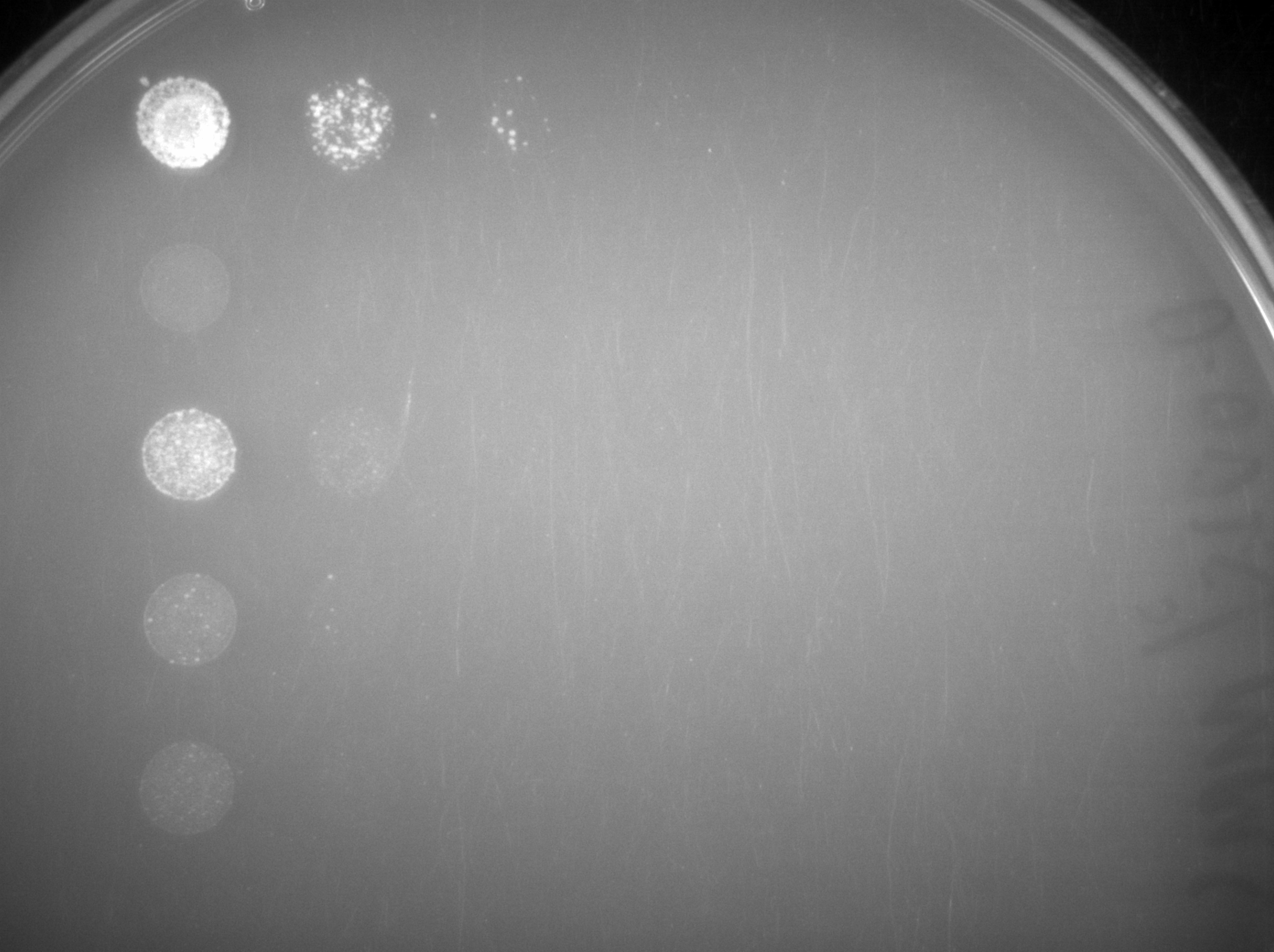

Supplement: Supplementary file 8 — Source data Fig. 3 [file 44318_2024_139_MOESM8_ESM.zip › Fig 3 data/Figure3E_data/Sgs1 rejector sensitivity-mec1/00025mms.jpg]

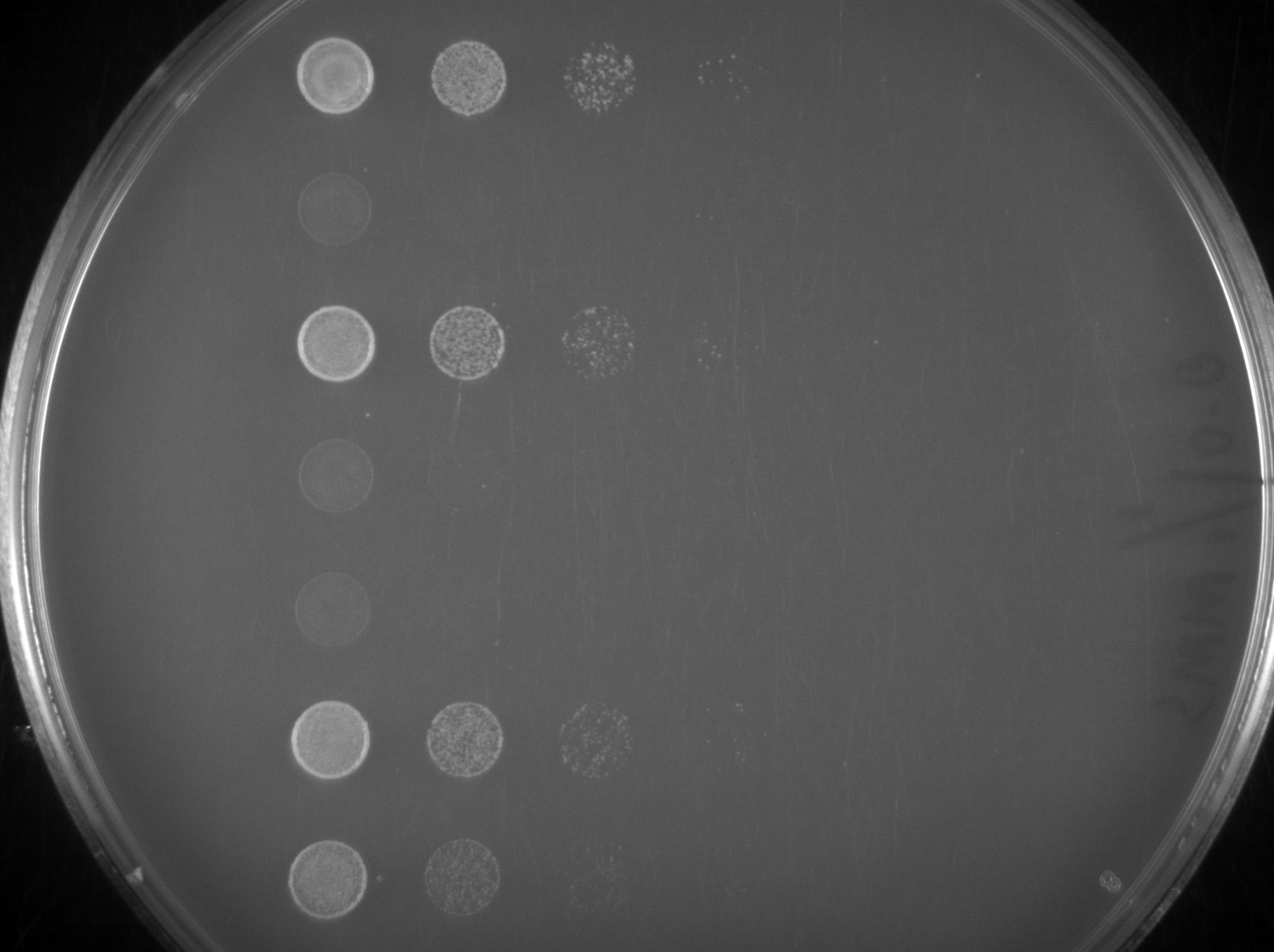

Supplement: Supplementary file 8 — Source data Fig. 3 [file 44318_2024_139_MOESM8_ESM.zip › Fig 3 data/Figure3E_data/Sgs1 rejector sensitivity-WT/001mms.jpg]

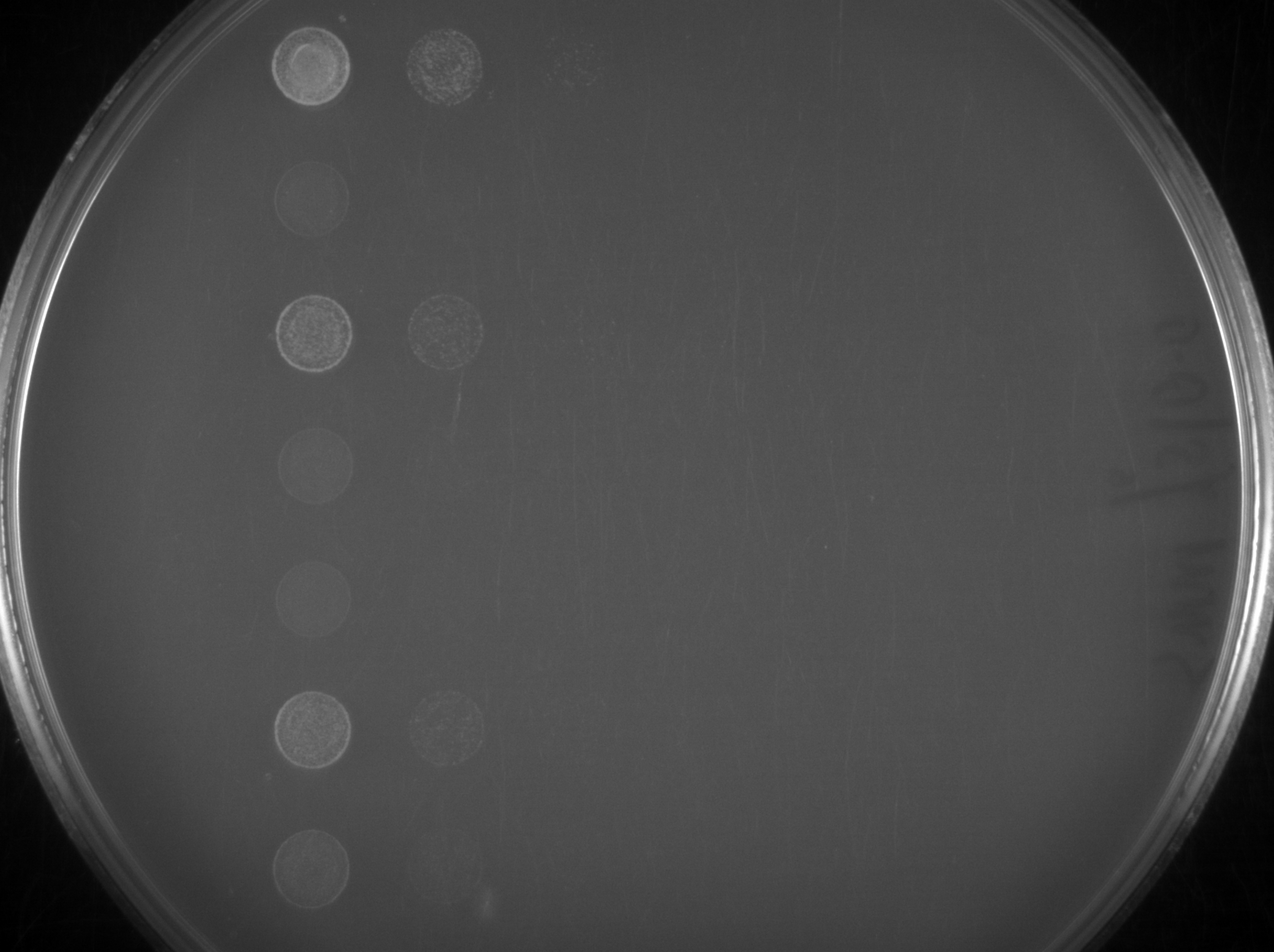

Supplement: Supplementary file 8 — Source data Fig. 3 [file 44318_2024_139_MOESM8_ESM.zip › Fig 3 data/Figure3E_data/Sgs1 rejector sensitivity-WT/0015mms.jpg]

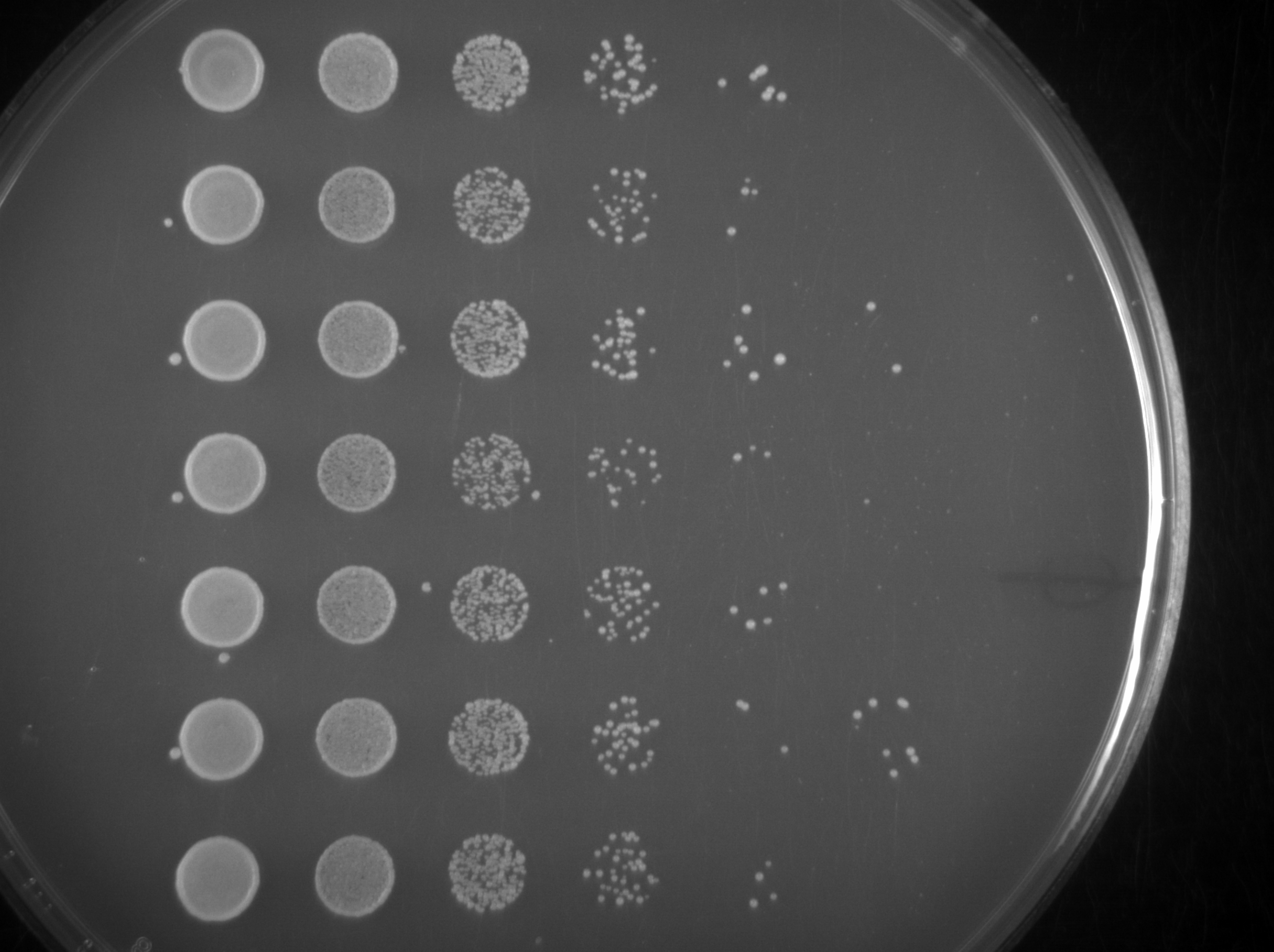

Supplement: Supplementary file 8 — Source data Fig. 3 [file 44318_2024_139_MOESM8_ESM.zip › Fig 3 data/Figure3E_data/Sgs1 rejector sensitivity-WT/untreated.jpg]

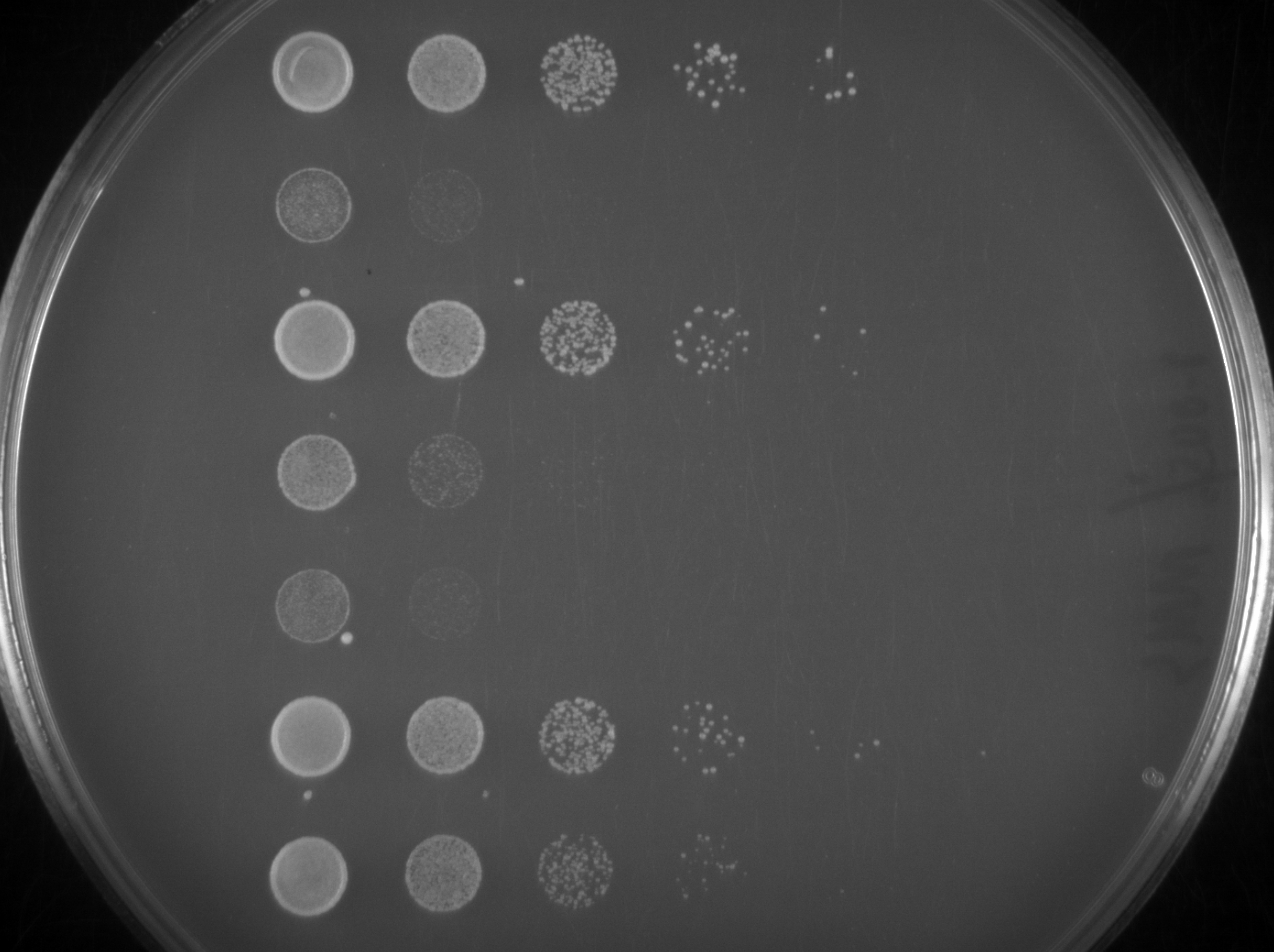

Supplement: Supplementary file 8 — Source data Fig. 3 [file 44318_2024_139_MOESM8_ESM.zip › Fig 3 data/Figure3E_data/Sgs1 rejector sensitivity-WT/0005mms.jpg]

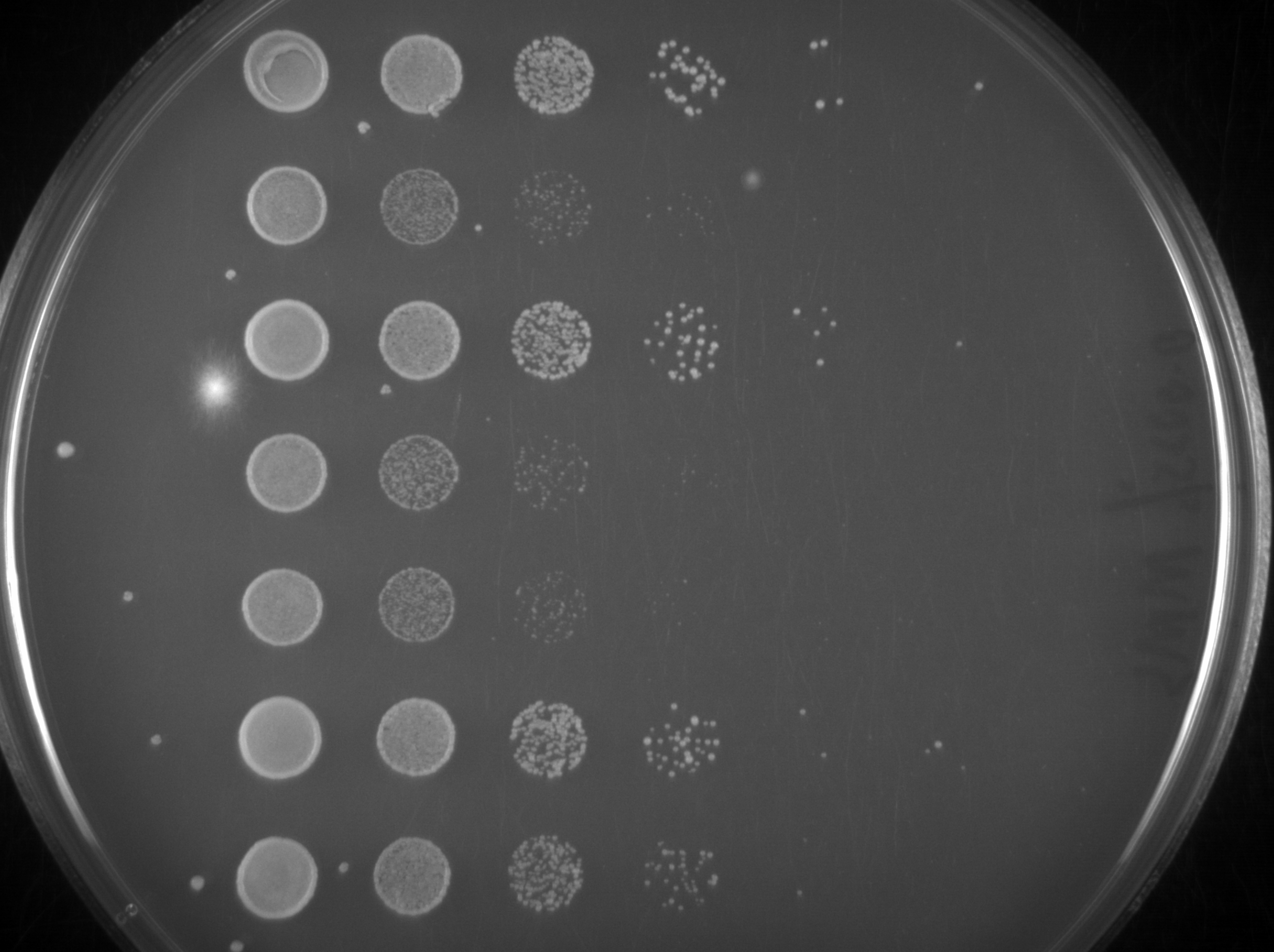

Supplement: Supplementary file 8 — Source data Fig. 3 [file 44318_2024_139_MOESM8_ESM.zip › Fig 3 data/Figure3E_data/Sgs1 rejector sensitivity-WT/00025mms.jpg]

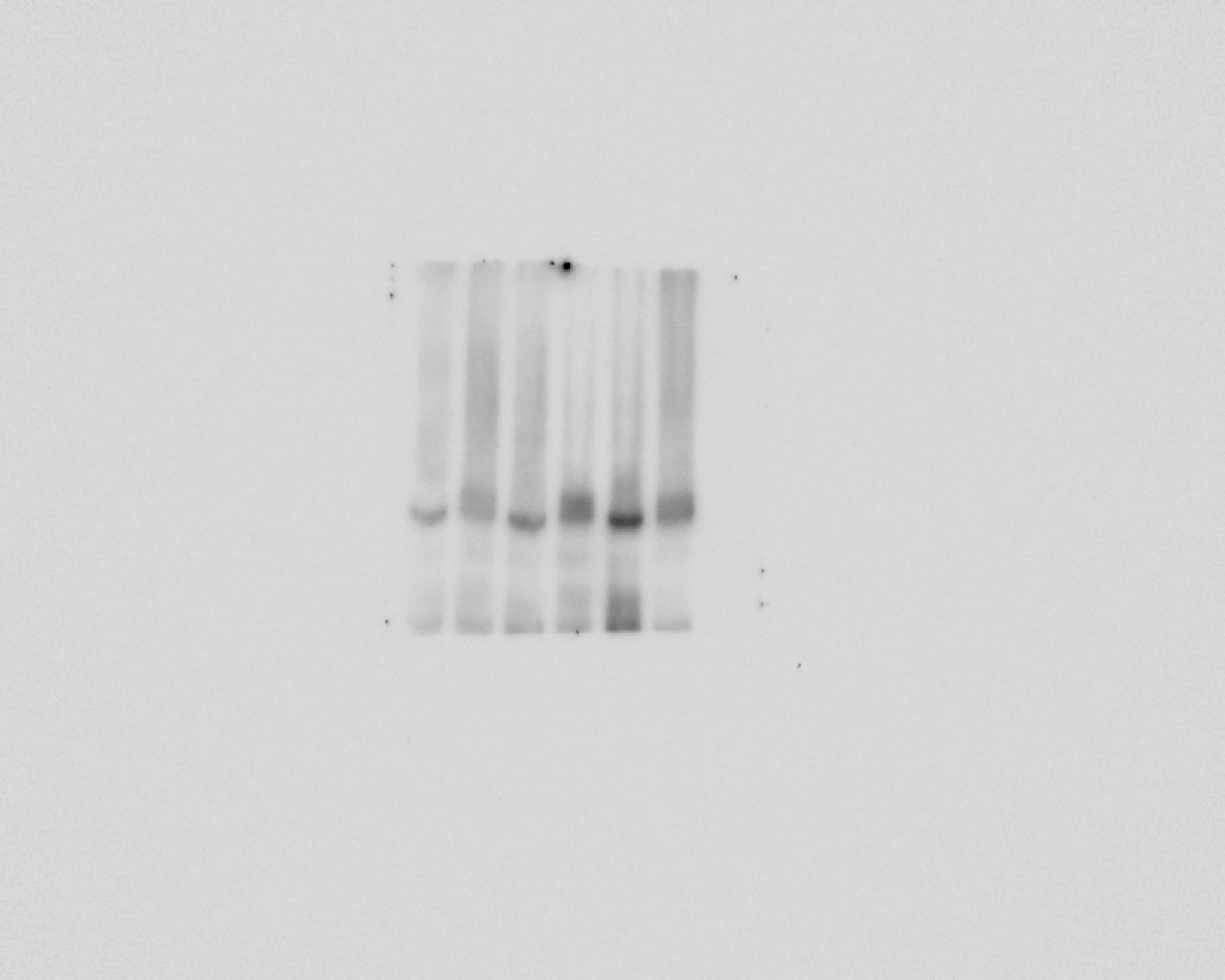

Supplement: Supplementary file 8 — Source data Fig. 3 [file 44318_2024_139_MOESM8_ESM.zip › Fig 3 data/Figure3H_data/Rad53_activation_rep2/Rad53 activation_rep1.png]

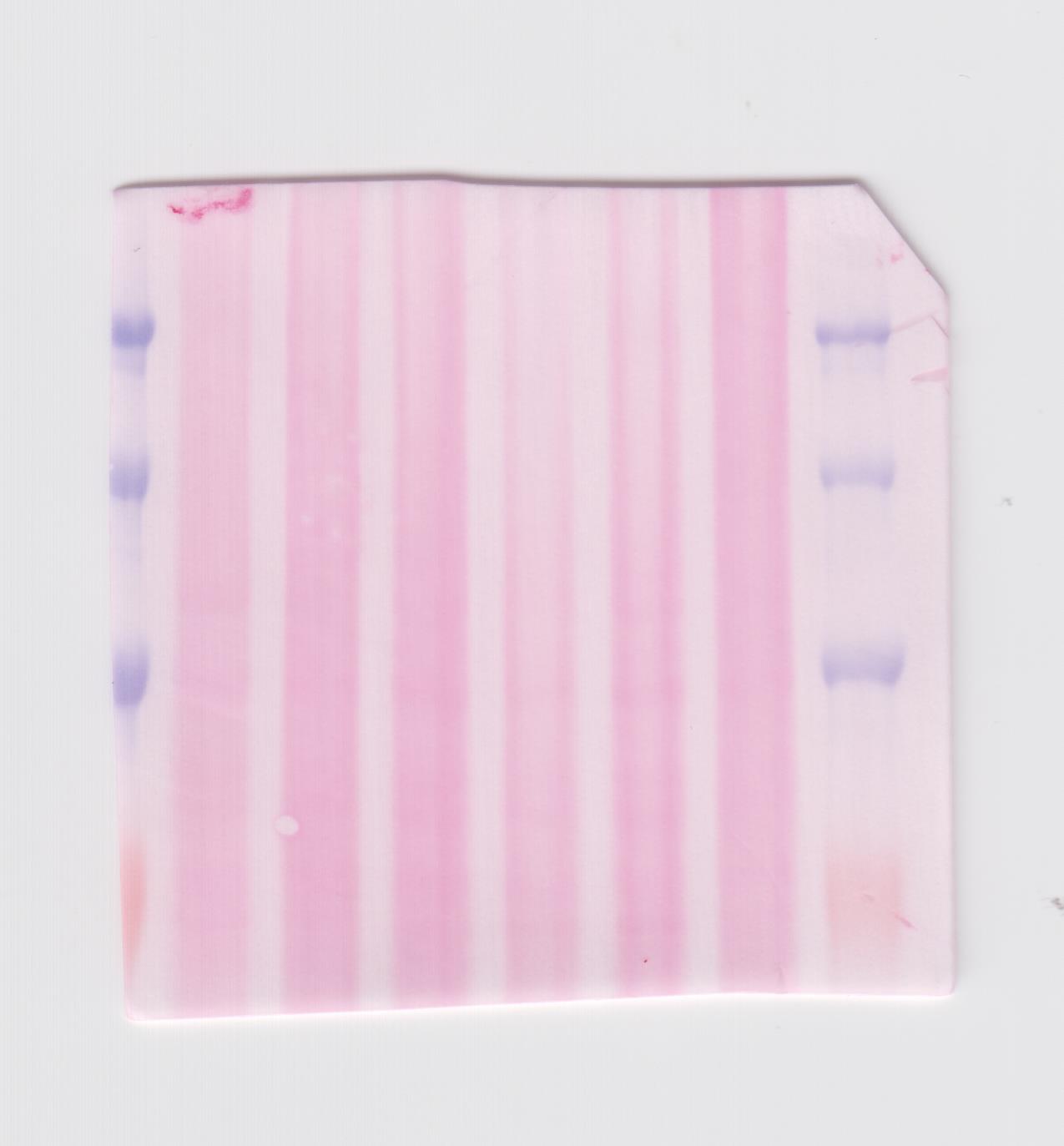

Supplement: Supplementary file 8 — Source data Fig. 3 [file 44318_2024_139_MOESM8_ESM.zip › Fig 3 data/Figure3H_data/Rad53_activation_rep2/Ponceau S.jpeg]

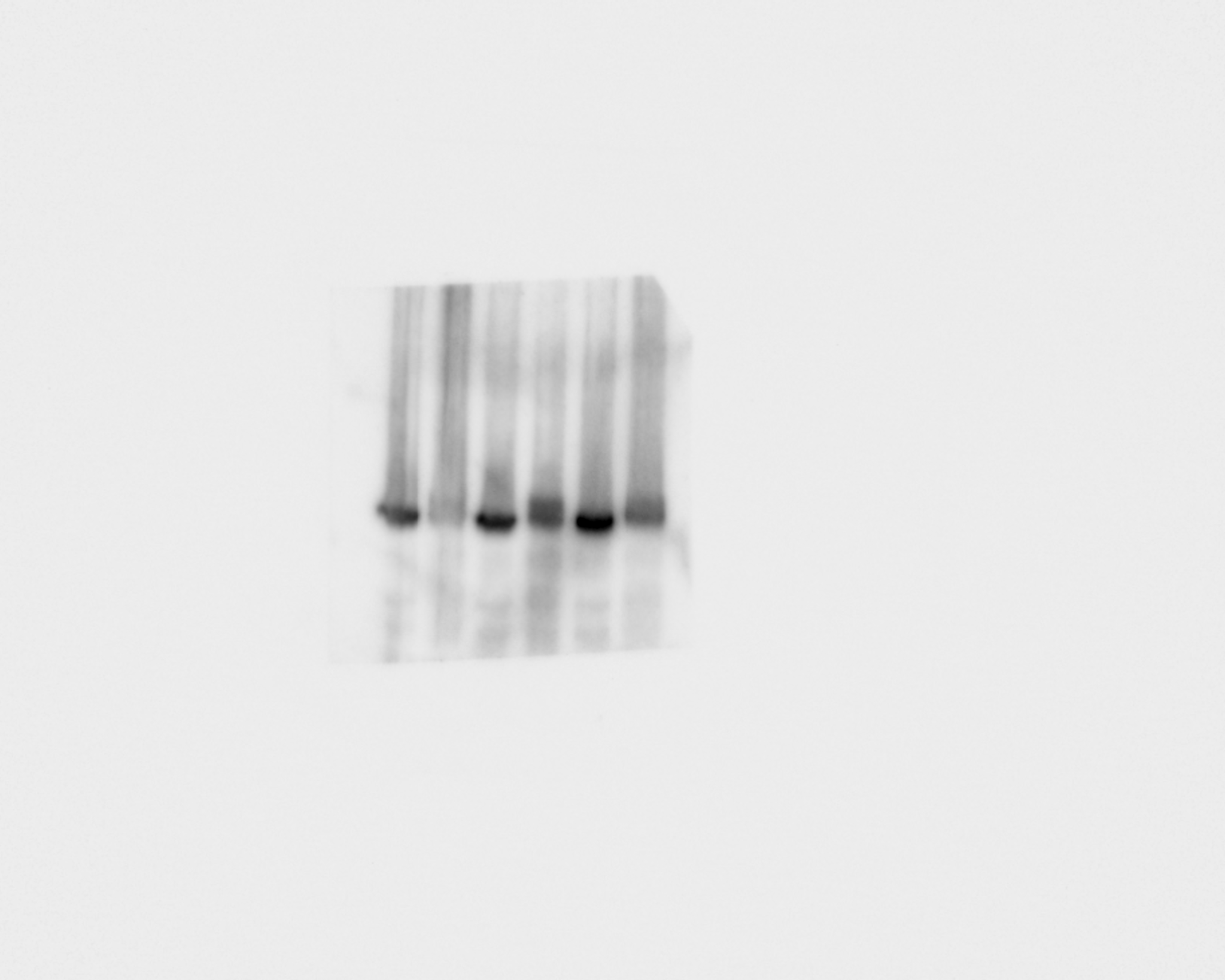

Supplement: Supplementary file 8 — Source data Fig. 3 [file 44318_2024_139_MOESM8_ESM.zip › Fig 3 data/Figure3H_data/Rad53_activation_rep1/rad53 activation_rep2.png]

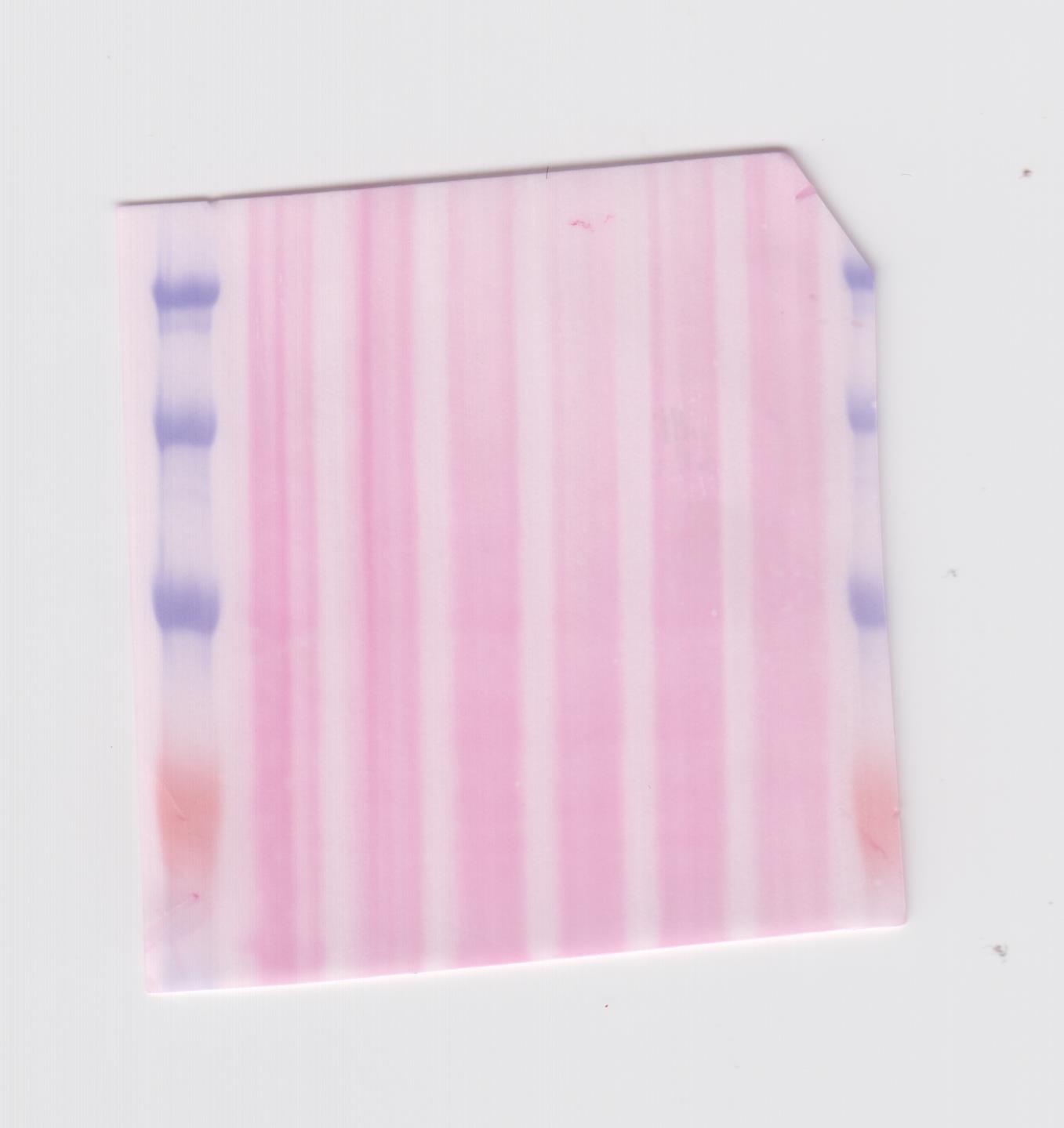

Supplement: Supplementary file 8 — Source data Fig. 3 [file 44318_2024_139_MOESM8_ESM.zip › Fig 3 data/Figure3H_data/Rad53_activation_rep1/Ponceau S_R.jpeg]

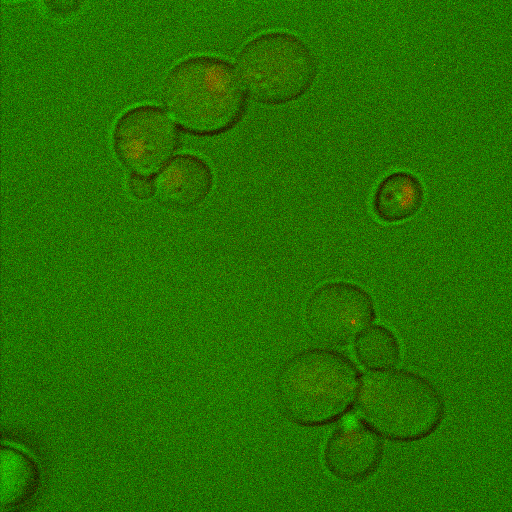

Supplement: Supplementary file 8 — Source data Fig. 3 [file 44318_2024_139_MOESM8_ESM.zip › Fig 3 data/Fig 3G_data/replicate 2/rad9del+sgs1+001MMS/13.tif]

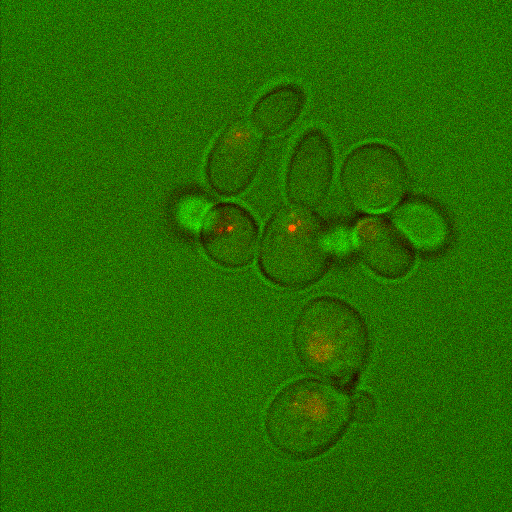

Supplement: Supplementary file 8 — Source data Fig. 3 [file 44318_2024_139_MOESM8_ESM.zip › Fig 3 data/Fig 3G_data/replicate 2/rad9del+sgs1+001MMS/12.tif]

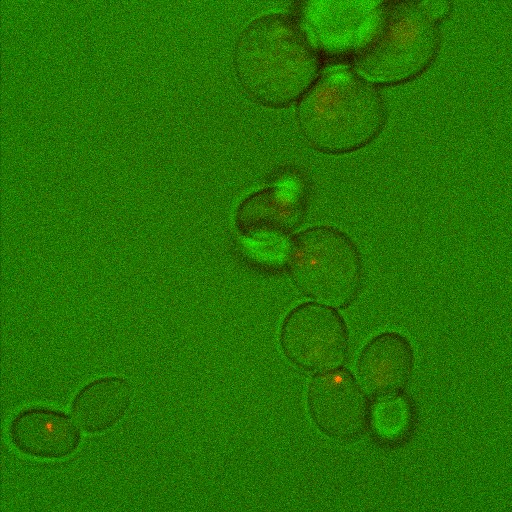

Supplement: Supplementary file 8 — Source data Fig. 3 [file 44318_2024_139_MOESM8_ESM.zip › Fig 3 data/Fig 3G_data/replicate 2/rad9del+sgs1+001MMS/10.tif]

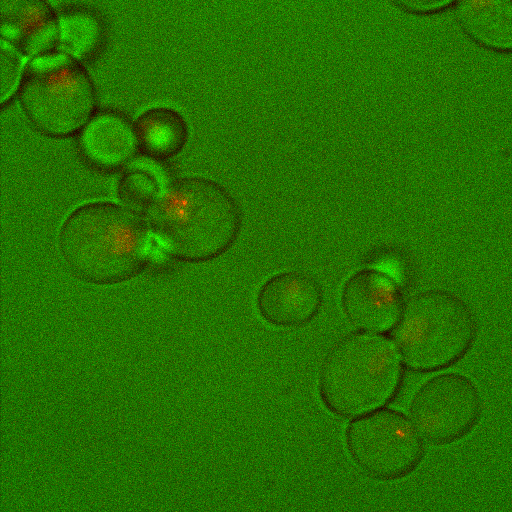

Supplement: Supplementary file 8 — Source data Fig. 3 [file 44318_2024_139_MOESM8_ESM.zip › Fig 3 data/Fig 3G_data/replicate 2/rad9del+sgs1+001MMS/11.tif]

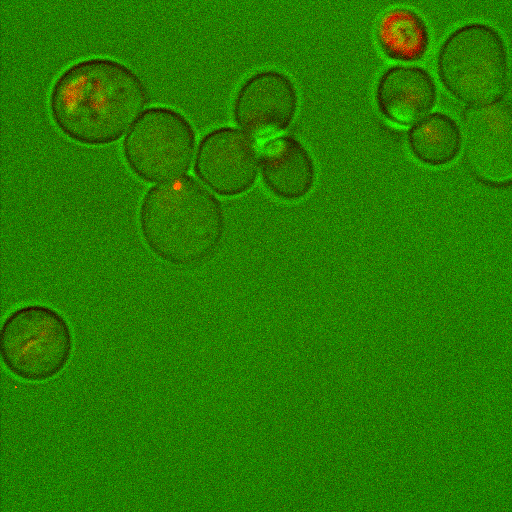

Supplement: Supplementary file 8 — Source data Fig. 3 [file 44318_2024_139_MOESM8_ESM.zip › Fig 3 data/Fig 3G_data/replicate 2/rad9del+sgs1+001MMS/15.tif]

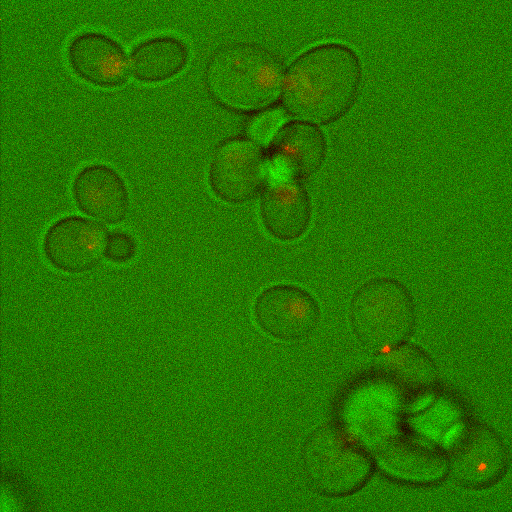

Supplement: Supplementary file 8 — Source data Fig. 3 [file 44318_2024_139_MOESM8_ESM.zip › Fig 3 data/Fig 3G_data/replicate 2/rad9del+sgs1+001MMS/14.tif]

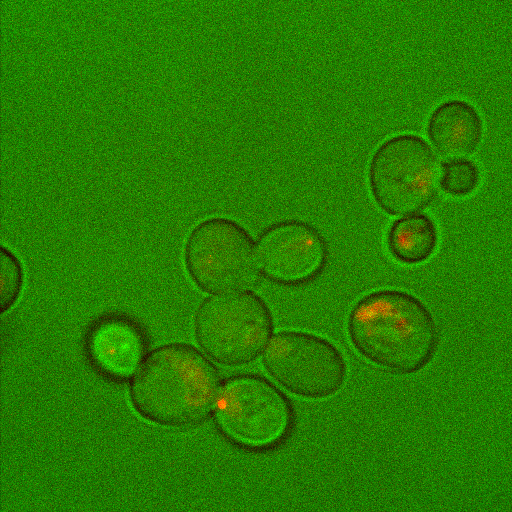

Supplement: Supplementary file 8 — Source data Fig. 3 [file 44318_2024_139_MOESM8_ESM.zip › Fig 3 data/Fig 3G_data/replicate 2/rad9del+sgs1+001MMS/16.tif]

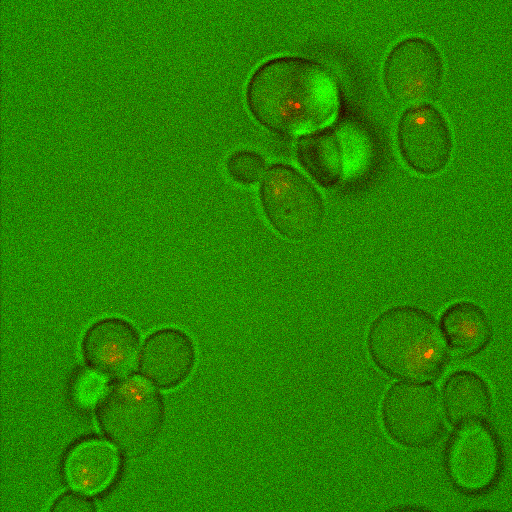

Supplement: Supplementary file 8 — Source data Fig. 3 [file 44318_2024_139_MOESM8_ESM.zip › Fig 3 data/Fig 3G_data/replicate 2/rad9del+sgs1+001MMS/17.tif]

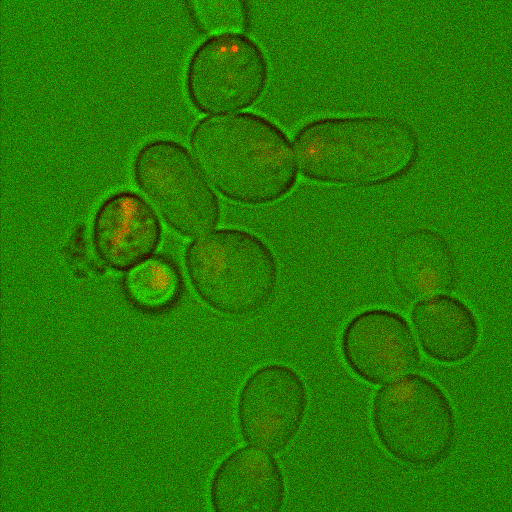

Supplement: Supplementary file 8 — Source data Fig. 3 [file 44318_2024_139_MOESM8_ESM.zip › Fig 3 data/Fig 3G_data/replicate 2/rad9del+sgs1+001MMS/9.tif]

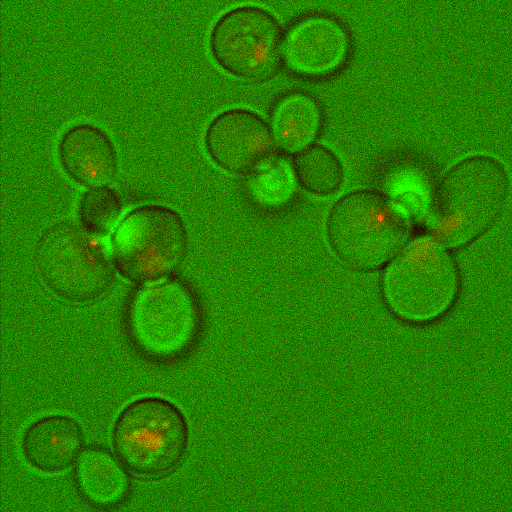

Supplement: Supplementary file 8 — Source data Fig. 3 [file 44318_2024_139_MOESM8_ESM.zip › Fig 3 data/Fig 3G_data/replicate 2/rad9del+sgs1+001MMS/8.tif]

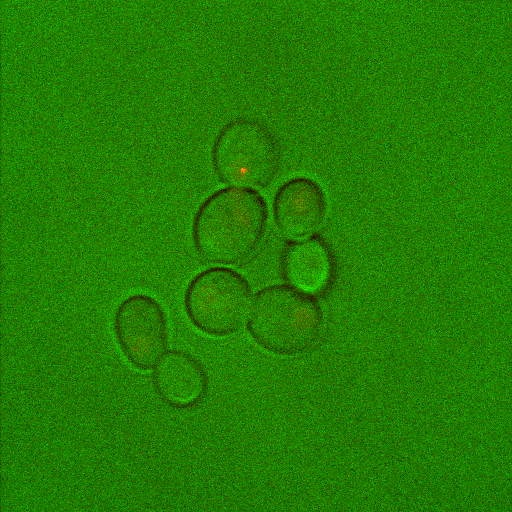

Supplement: Supplementary file 8 — Source data Fig. 3 [file 44318_2024_139_MOESM8_ESM.zip › Fig 3 data/Fig 3G_data/replicate 2/rad9del+sgs1+001MMS/3.tif]

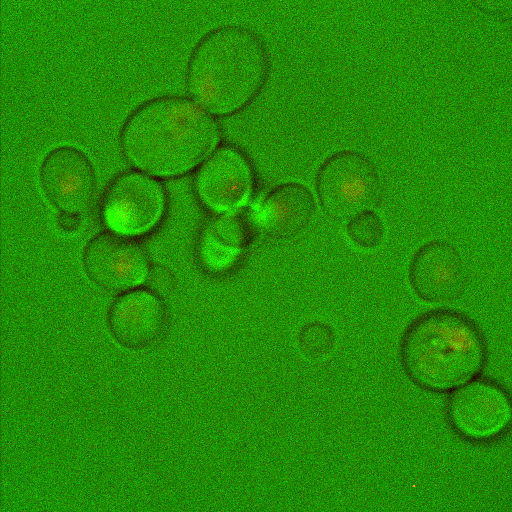

Supplement: Supplementary file 8 — Source data Fig. 3 [file 44318_2024_139_MOESM8_ESM.zip › Fig 3 data/Fig 3G_data/replicate 2/rad9del+sgs1+001MMS/2.tif]

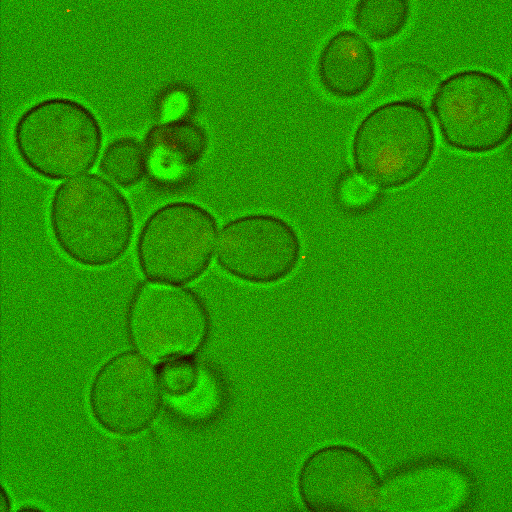

Supplement: Supplementary file 8 — Source data Fig. 3 [file 44318_2024_139_MOESM8_ESM.zip › Fig 3 data/Fig 3G_data/replicate 2/rad9del+sgs1+001MMS/1.tif]

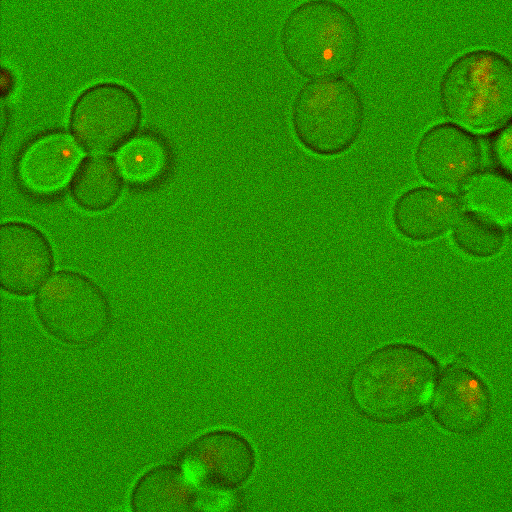

Supplement: Supplementary file 8 — Source data Fig. 3 [file 44318_2024_139_MOESM8_ESM.zip › Fig 3 data/Fig 3G_data/replicate 2/rad9del+sgs1+001MMS/5.tif]

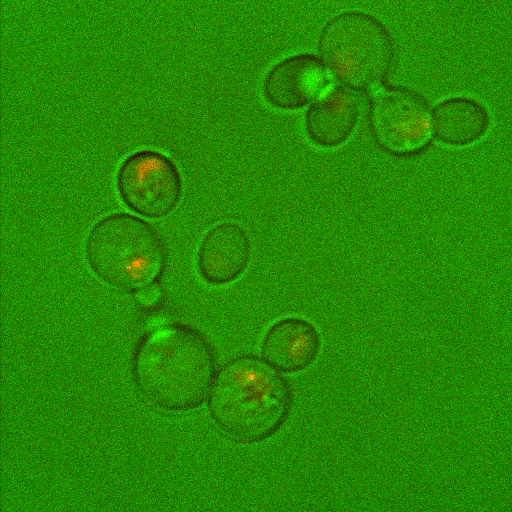

Supplement: Supplementary file 8 — Source data Fig. 3 [file 44318_2024_139_MOESM8_ESM.zip › Fig 3 data/Fig 3G_data/replicate 2/rad9del+sgs1+001MMS/4.tif]

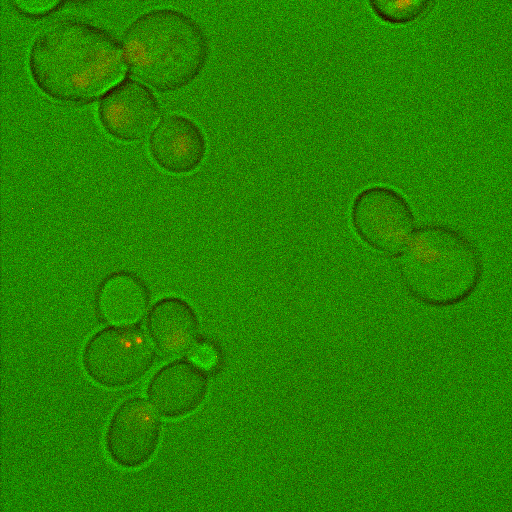

Supplement: Supplementary file 8 — Source data Fig. 3 [file 44318_2024_139_MOESM8_ESM.zip › Fig 3 data/Fig 3G_data/replicate 2/rad9del+sgs1+001MMS/6.tif]

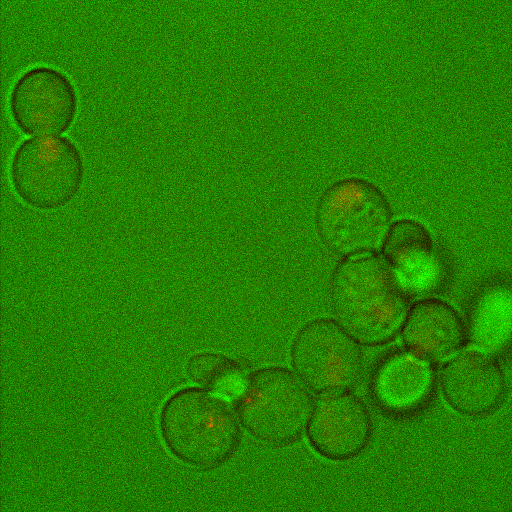

Supplement: Supplementary file 8 — Source data Fig. 3 [file 44318_2024_139_MOESM8_ESM.zip › Fig 3 data/Fig 3G_data/replicate 2/rad9del+sgs1+001MMS/7.tif]

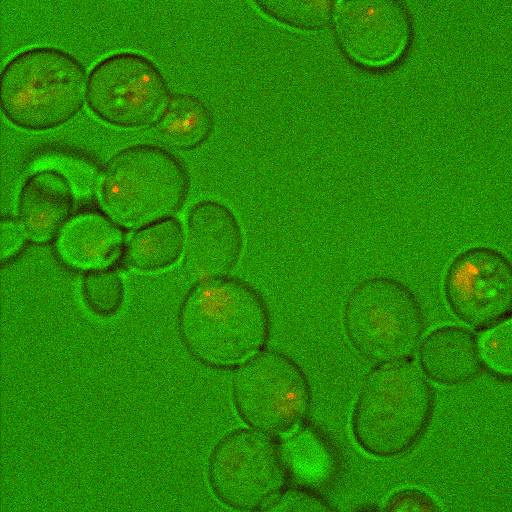

Supplement: Supplementary file 8 — Source data Fig. 3 [file 44318_2024_139_MOESM8_ESM.zip › Fig 3 data/Fig 3G_data/replicate 2/rad9del+sgs1+001MMS/19.tif]

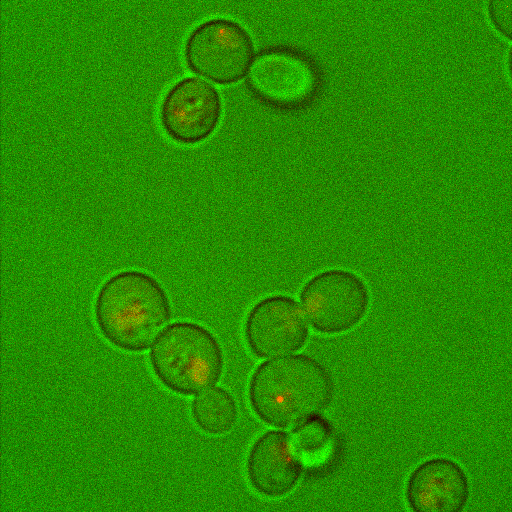

Supplement: Supplementary file 8 — Source data Fig. 3 [file 44318_2024_139_MOESM8_ESM.zip › Fig 3 data/Fig 3G_data/replicate 2/rad9del+sgs1+001MMS/18.tif]

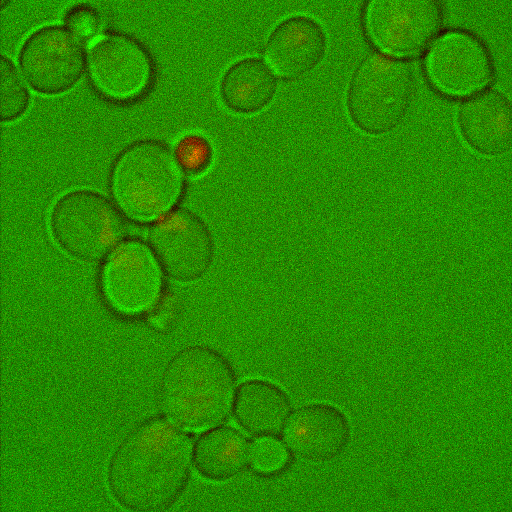

Supplement: Supplementary file 8 — Source data Fig. 3 [file 44318_2024_139_MOESM8_ESM.zip › Fig 3 data/Fig 3G_data/replicate 2/rad9del+sgs1+001MMS/20.tif]

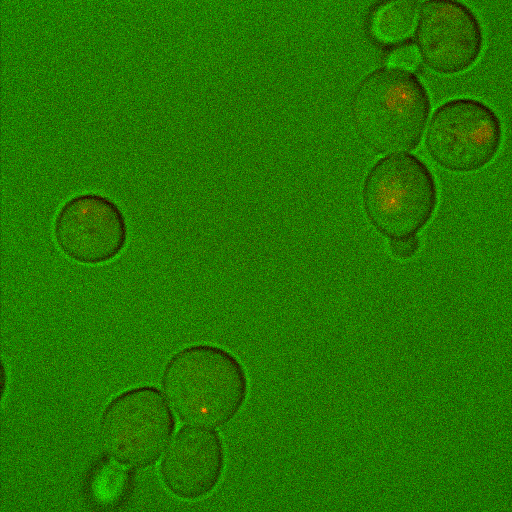

Supplement: Supplementary file 8 — Source data Fig. 3 [file 44318_2024_139_MOESM8_ESM.zip › Fig 3 data/Fig 3G_data/replicate 2/rad9del+D2-sgs1+001MMS/13.tif]

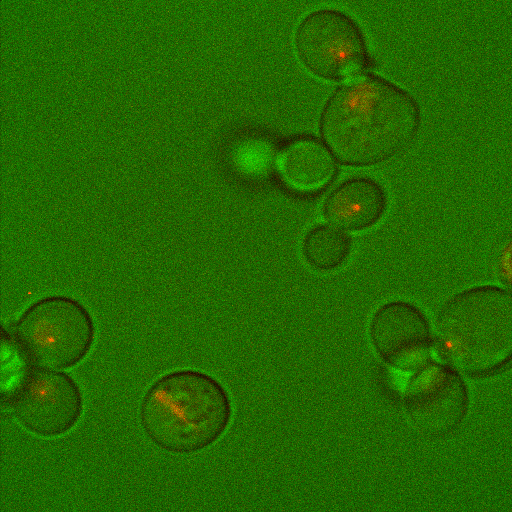

Supplement: Supplementary file 8 — Source data Fig. 3 [file 44318_2024_139_MOESM8_ESM.zip › Fig 3 data/Fig 3G_data/replicate 2/rad9del+D2-sgs1+001MMS/12.tif]

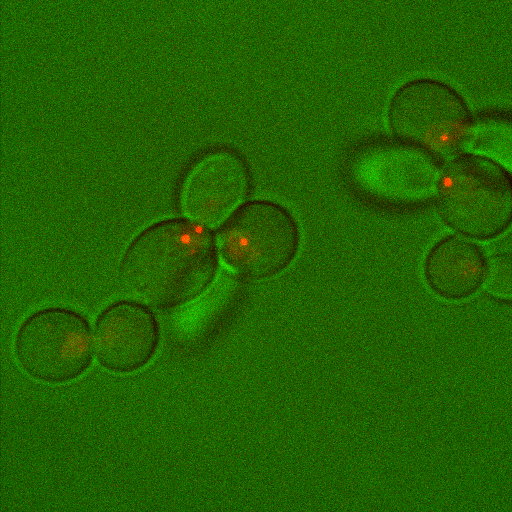

Supplement: Supplementary file 8 — Source data Fig. 3 [file 44318_2024_139_MOESM8_ESM.zip › Fig 3 data/Fig 3G_data/replicate 2/rad9del+D2-sgs1+001MMS/10.tif]

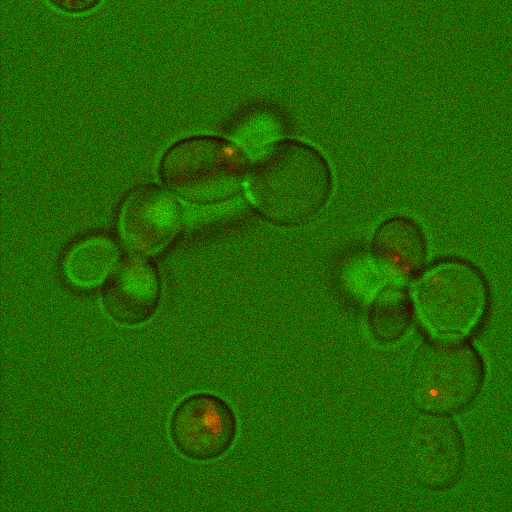

Supplement: Supplementary file 8 — Source data Fig. 3 [file 44318_2024_139_MOESM8_ESM.zip › Fig 3 data/Fig 3G_data/replicate 2/rad9del+D2-sgs1+001MMS/11.tif]

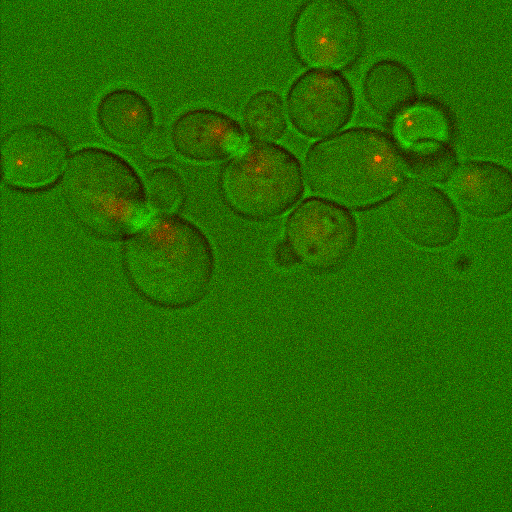

Supplement: Supplementary file 8 — Source data Fig. 3 [file 44318_2024_139_MOESM8_ESM.zip › Fig 3 data/Fig 3G_data/replicate 2/rad9del+D2-sgs1+001MMS/15.tif]

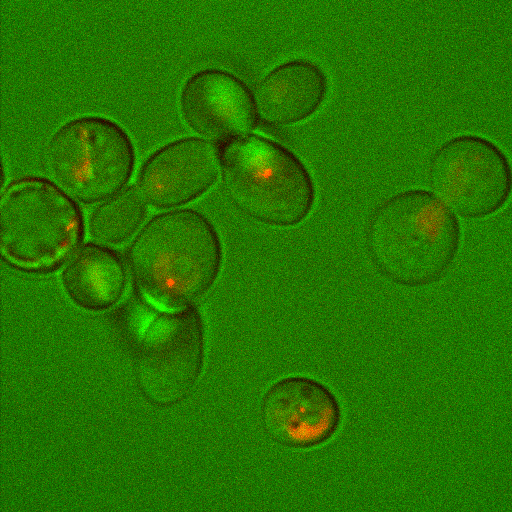

Supplement: Supplementary file 8 — Source data Fig. 3 [file 44318_2024_139_MOESM8_ESM.zip › Fig 3 data/Fig 3G_data/replicate 2/rad9del+D2-sgs1+001MMS/14.tif]

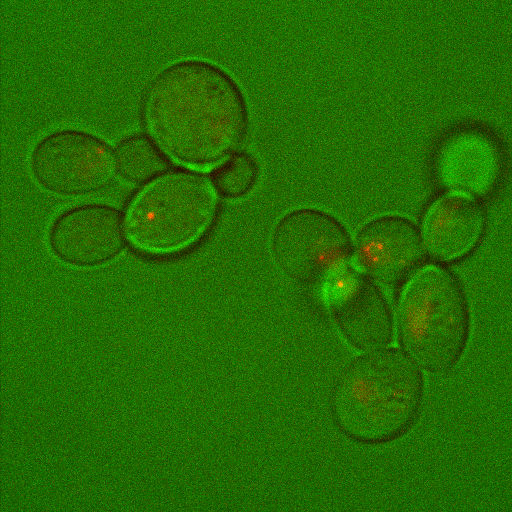

Supplement: Supplementary file 8 — Source data Fig. 3 [file 44318_2024_139_MOESM8_ESM.zip › Fig 3 data/Fig 3G_data/replicate 2/rad9del+D2-sgs1+001MMS/16.tif]

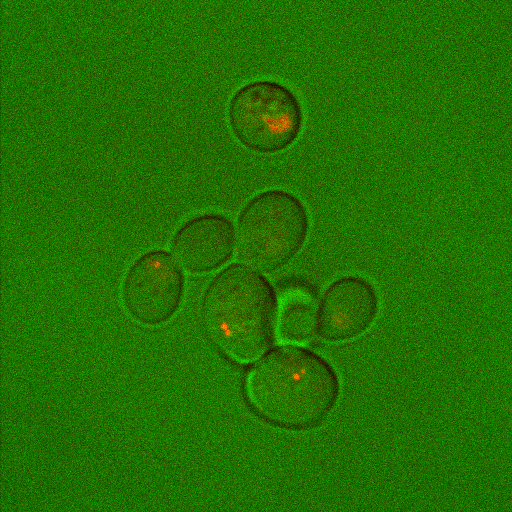

Supplement: Supplementary file 8 — Source data Fig. 3 [file 44318_2024_139_MOESM8_ESM.zip › Fig 3 data/Fig 3G_data/replicate 2/rad9del+D2-sgs1+001MMS/17.tif]

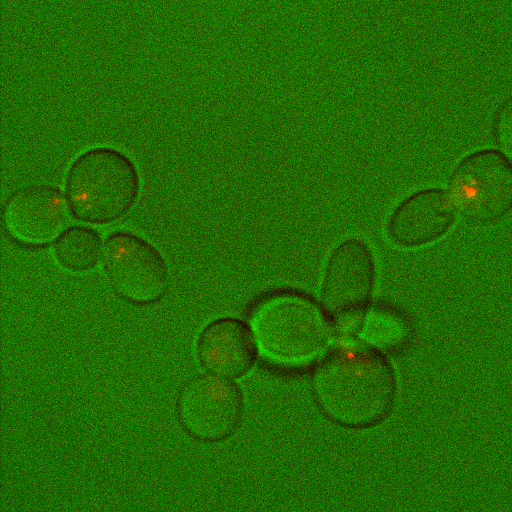

Supplement: Supplementary file 8 — Source data Fig. 3 [file 44318_2024_139_MOESM8_ESM.zip › Fig 3 data/Fig 3G_data/replicate 2/rad9del+D2-sgs1+001MMS/9.tif]

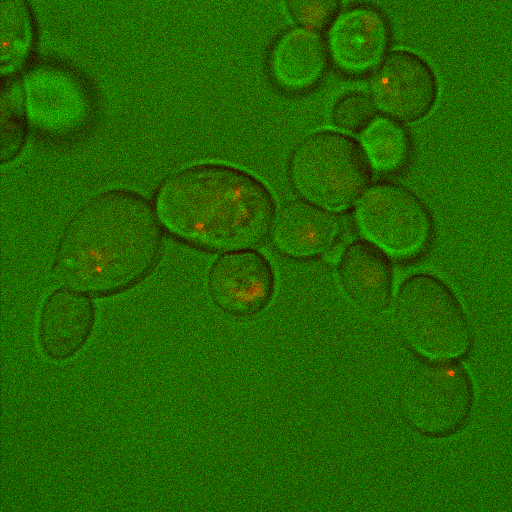

Supplement: Supplementary file 8 — Source data Fig. 3 [file 44318_2024_139_MOESM8_ESM.zip › Fig 3 data/Fig 3G_data/replicate 2/rad9del+D2-sgs1+001MMS/8.tif]

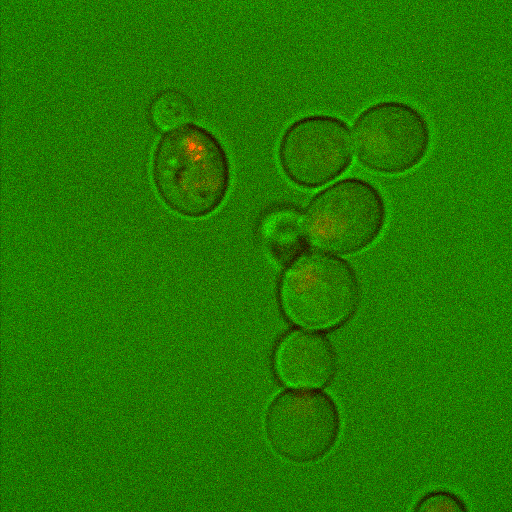

Supplement: Supplementary file 8 — Source data Fig. 3 [file 44318_2024_139_MOESM8_ESM.zip › Fig 3 data/Fig 3G_data/replicate 2/rad9del+D2-sgs1+001MMS/3.tif]

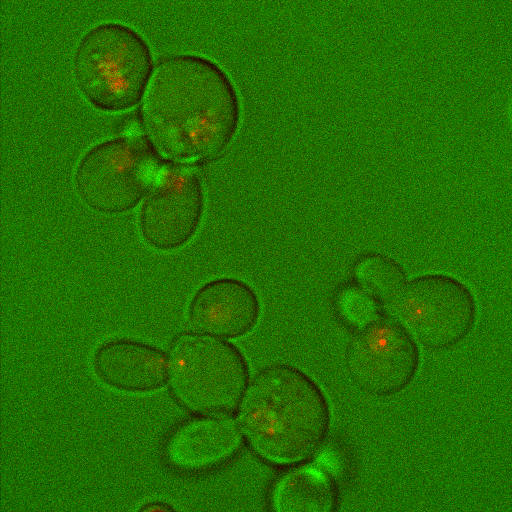

Supplement: Supplementary file 8 — Source data Fig. 3 [file 44318_2024_139_MOESM8_ESM.zip › Fig 3 data/Fig 3G_data/replicate 2/rad9del+D2-sgs1+001MMS/2.tif]

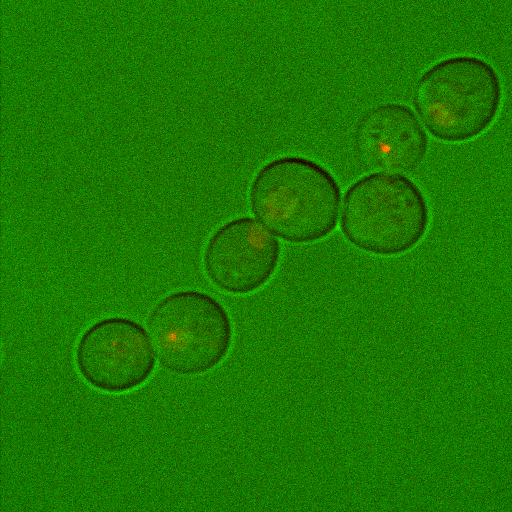

Supplement: Supplementary file 8 — Source data Fig. 3 [file 44318_2024_139_MOESM8_ESM.zip › Fig 3 data/Fig 3G_data/replicate 2/rad9del+D2-sgs1+001MMS/1.tif]

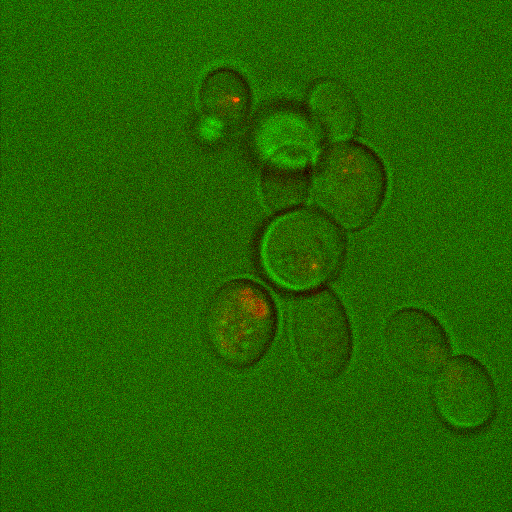

Supplement: Supplementary file 8 — Source data Fig. 3 [file 44318_2024_139_MOESM8_ESM.zip › Fig 3 data/Fig 3G_data/replicate 2/rad9del+D2-sgs1+001MMS/5.tif]

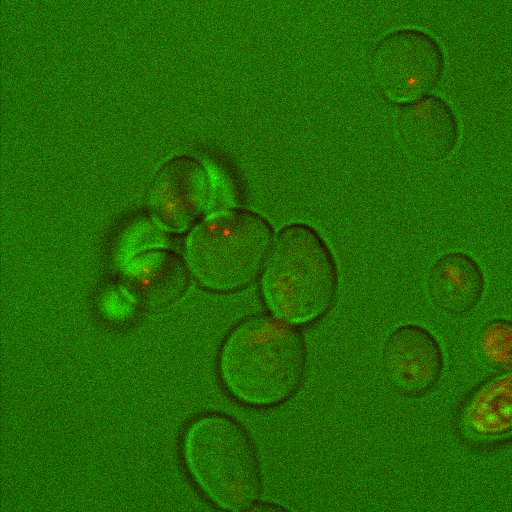

Supplement: Supplementary file 8 — Source data Fig. 3 [file 44318_2024_139_MOESM8_ESM.zip › Fig 3 data/Fig 3G_data/replicate 2/rad9del+D2-sgs1+001MMS/4.tif]

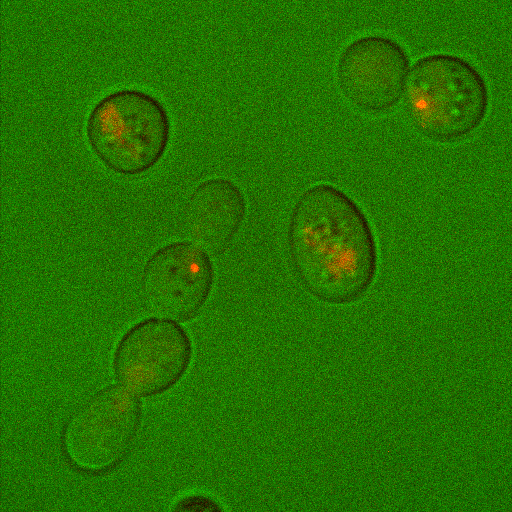

Supplement: Supplementary file 8 — Source data Fig. 3 [file 44318_2024_139_MOESM8_ESM.zip › Fig 3 data/Fig 3G_data/replicate 2/rad9del+D2-sgs1+001MMS/6.tif]

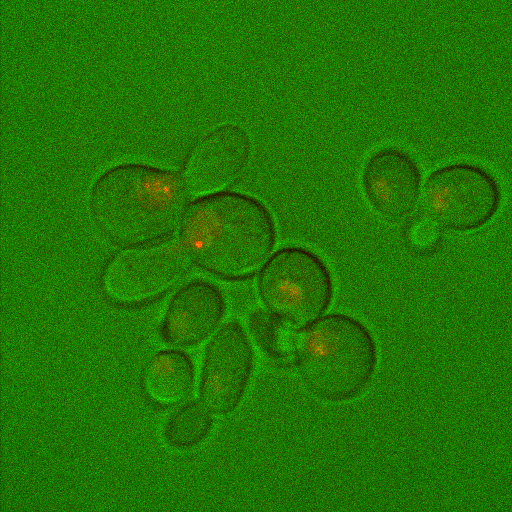

Supplement: Supplementary file 8 — Source data Fig. 3 [file 44318_2024_139_MOESM8_ESM.zip › Fig 3 data/Fig 3G_data/replicate 2/rad9del+D2-sgs1+001MMS/7.tif]

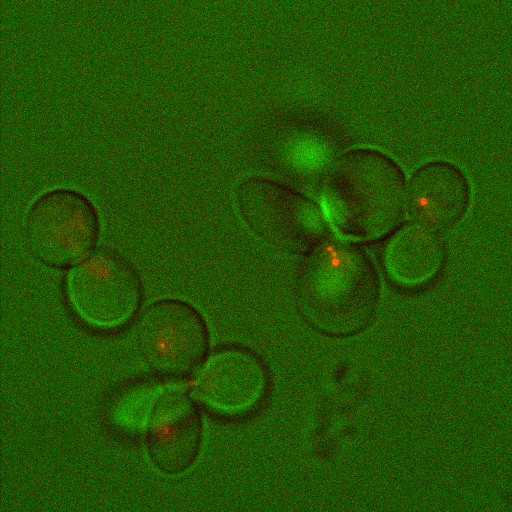

Supplement: Supplementary file 8 — Source data Fig. 3 [file 44318_2024_139_MOESM8_ESM.zip › Fig 3 data/Fig 3G_data/replicate 2/rad9del+D2-sgs1+001MMS/19.tif]

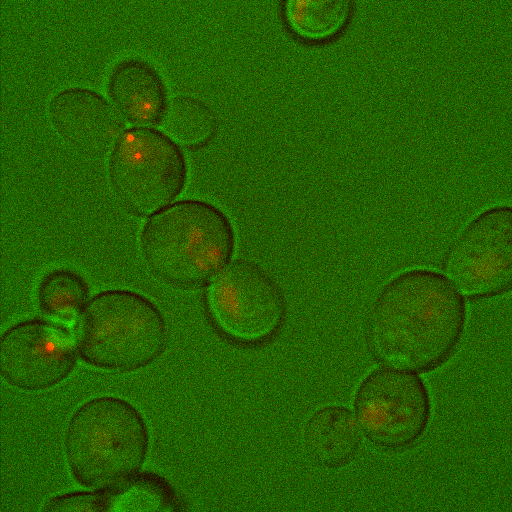

Supplement: Supplementary file 8 — Source data Fig. 3 [file 44318_2024_139_MOESM8_ESM.zip › Fig 3 data/Fig 3G_data/replicate 2/rad9del+D2-sgs1+001MMS/18.tif]

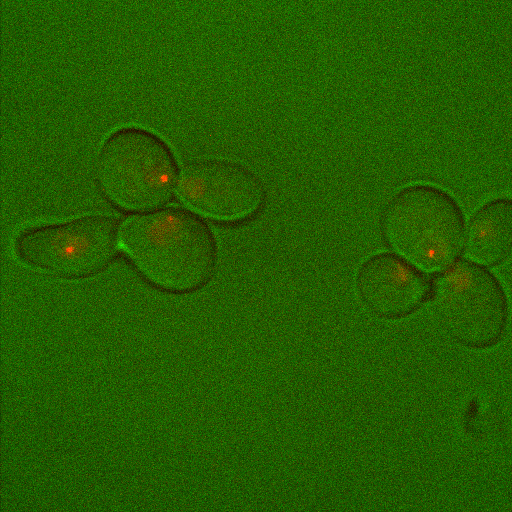

Supplement: Supplementary file 8 — Source data Fig. 3 [file 44318_2024_139_MOESM8_ESM.zip › Fig 3 data/Fig 3G_data/replicate 2/rad9del+D2-sgs1+001MMS/20.tif]

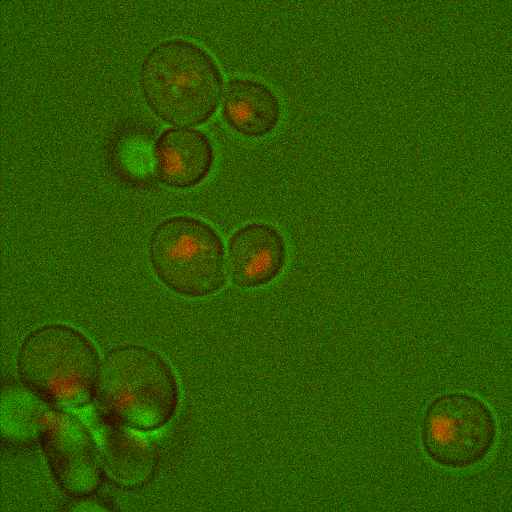

Supplement: Supplementary file 8 — Source data Fig. 3 [file 44318_2024_139_MOESM8_ESM.zip › Fig 3 data/Fig 3G_data/replicate 2/rad9del+sgs1/13.tif]

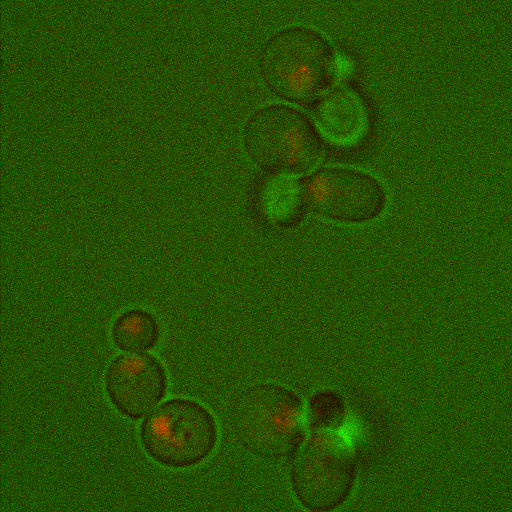

Supplement: Supplementary file 8 — Source data Fig. 3 [file 44318_2024_139_MOESM8_ESM.zip › Fig 3 data/Fig 3G_data/replicate 2/rad9del+sgs1/12.tif]

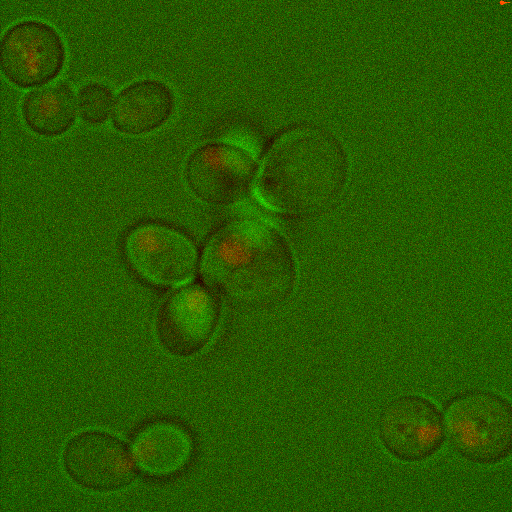

Supplement: Supplementary file 8 — Source data Fig. 3 [file 44318_2024_139_MOESM8_ESM.zip › Fig 3 data/Fig 3G_data/replicate 2/rad9del+sgs1/10.tif]

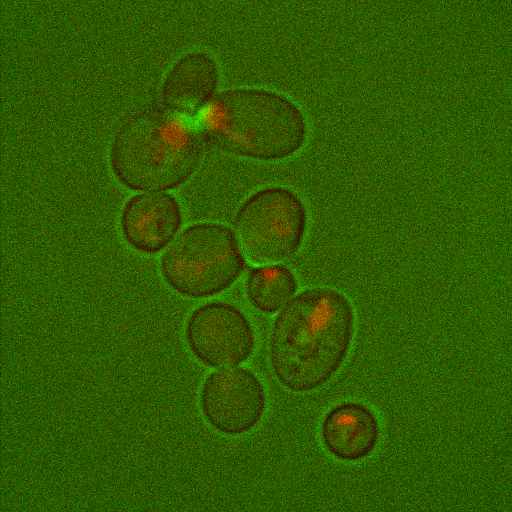

Supplement: Supplementary file 8 — Source data Fig. 3 [file 44318_2024_139_MOESM8_ESM.zip › Fig 3 data/Fig 3G_data/replicate 2/rad9del+sgs1/11.tif]

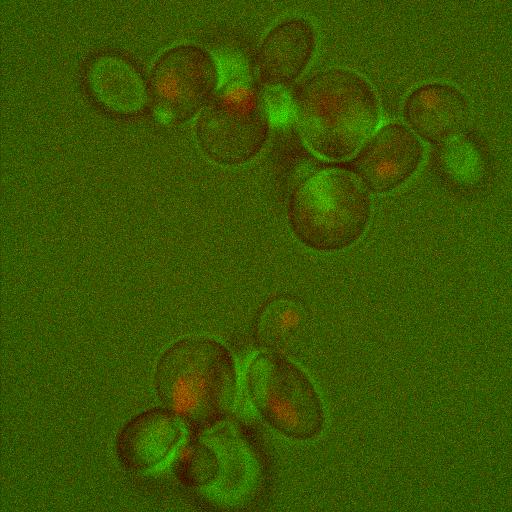

Supplement: Supplementary file 8 — Source data Fig. 3 [file 44318_2024_139_MOESM8_ESM.zip › Fig 3 data/Fig 3G_data/replicate 2/rad9del+sgs1/15.tif]

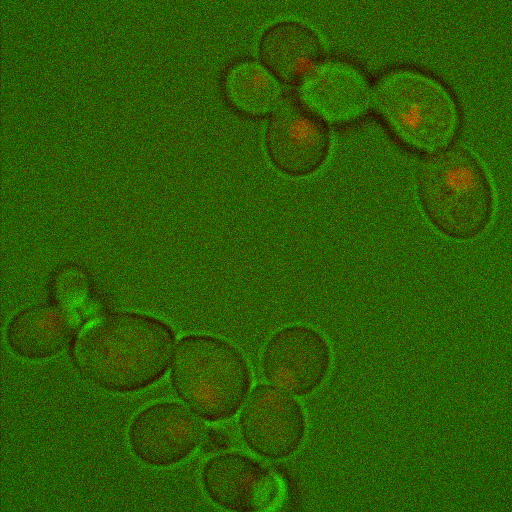

Supplement: Supplementary file 8 — Source data Fig. 3 [file 44318_2024_139_MOESM8_ESM.zip › Fig 3 data/Fig 3G_data/replicate 2/rad9del+sgs1/14.tif]

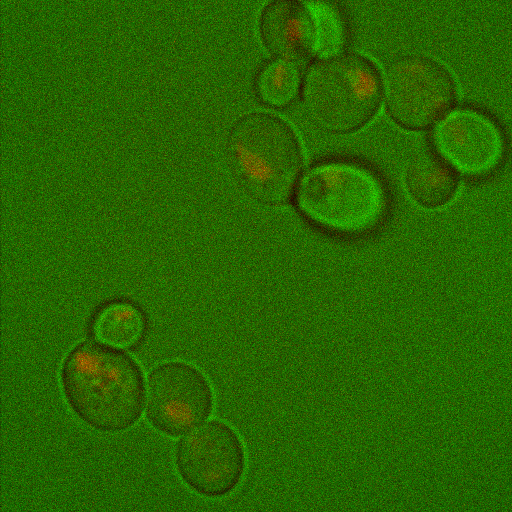

Supplement: Supplementary file 8 — Source data Fig. 3 [file 44318_2024_139_MOESM8_ESM.zip › Fig 3 data/Fig 3G_data/replicate 2/rad9del+sgs1/16.tif]

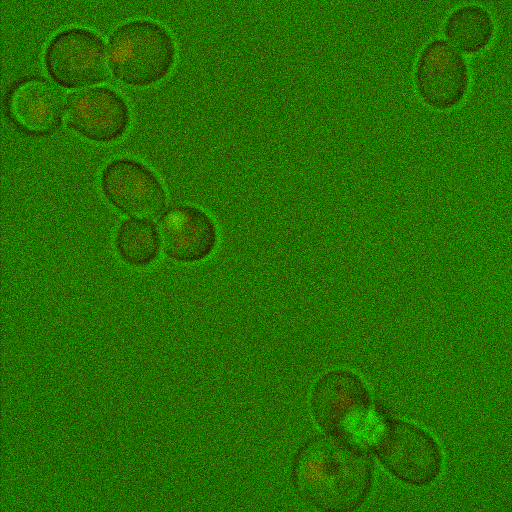

Supplement: Supplementary file 8 — Source data Fig. 3 [file 44318_2024_139_MOESM8_ESM.zip › Fig 3 data/Fig 3G_data/replicate 2/rad9del+sgs1/17.tif]

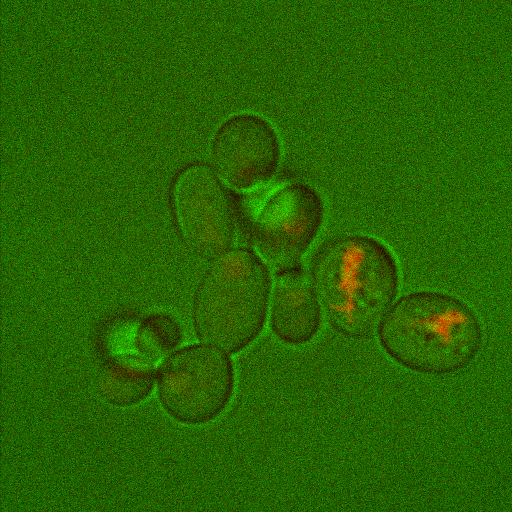

Supplement: Supplementary file 8 — Source data Fig. 3 [file 44318_2024_139_MOESM8_ESM.zip › Fig 3 data/Fig 3G_data/replicate 2/rad9del+sgs1/9.tif]

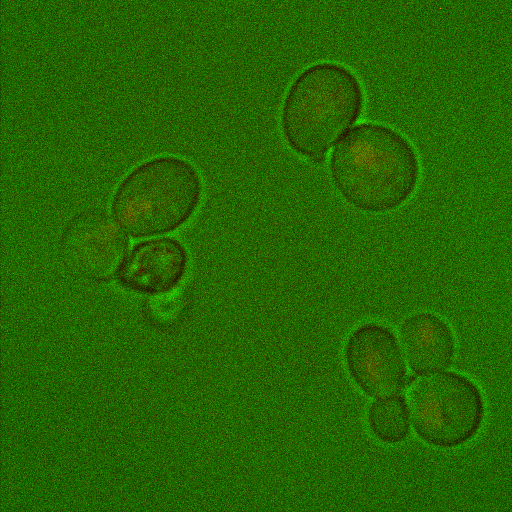

Supplement: Supplementary file 8 — Source data Fig. 3 [file 44318_2024_139_MOESM8_ESM.zip › Fig 3 data/Fig 3G_data/replicate 2/rad9del+sgs1/8.tif]

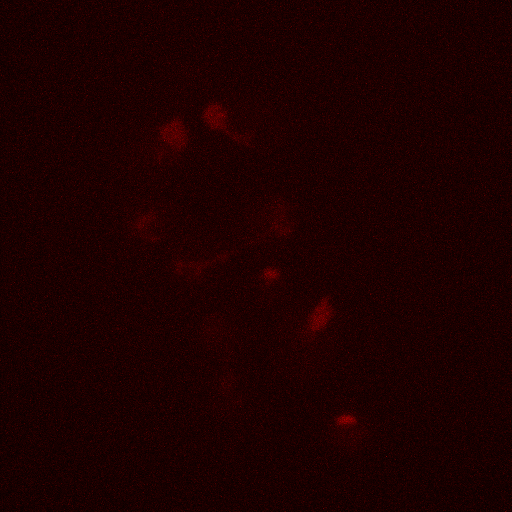

Supplement: Supplementary file 8 — Source data Fig. 3 [file 44318_2024_139_MOESM8_ESM.zip › Fig 3 data/Fig 3G_data/replicate 2/rad9del+sgs1/rep_image_Rad52.tif]

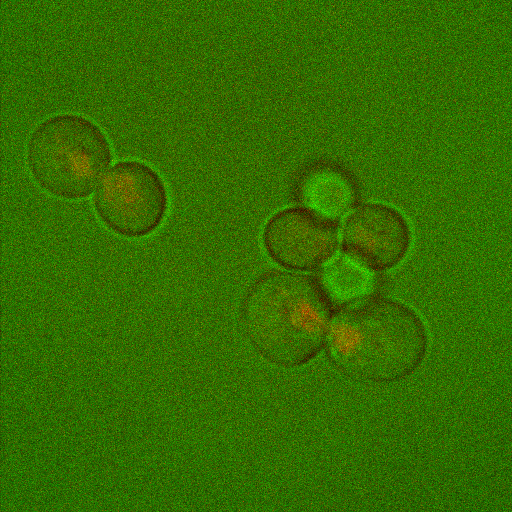

Supplement: Supplementary file 8 — Source data Fig. 3 [file 44318_2024_139_MOESM8_ESM.zip › Fig 3 data/Fig 3G_data/replicate 2/rad9del+sgs1/3.tif]

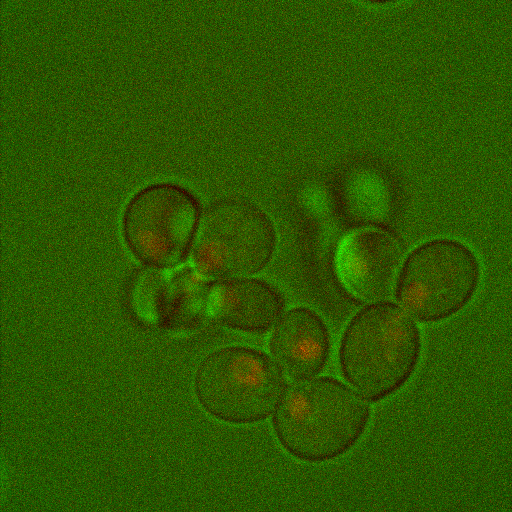

Supplement: Supplementary file 8 — Source data Fig. 3 [file 44318_2024_139_MOESM8_ESM.zip › Fig 3 data/Fig 3G_data/replicate 2/rad9del+sgs1/2.tif]

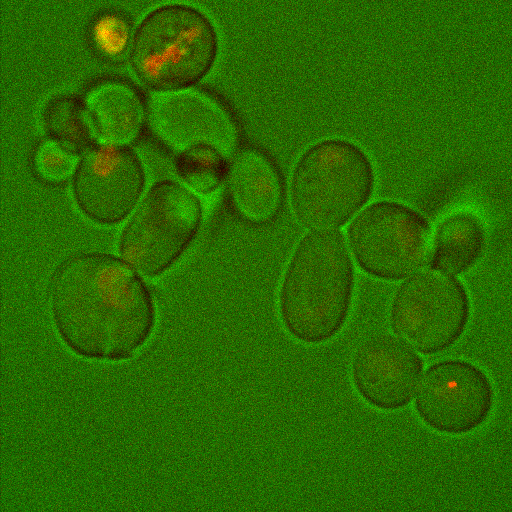

Supplement: Supplementary file 8 — Source data Fig. 3 [file 44318_2024_139_MOESM8_ESM.zip › Fig 3 data/Fig 3G_data/replicate 2/rad9del+sgs1/1.tif]

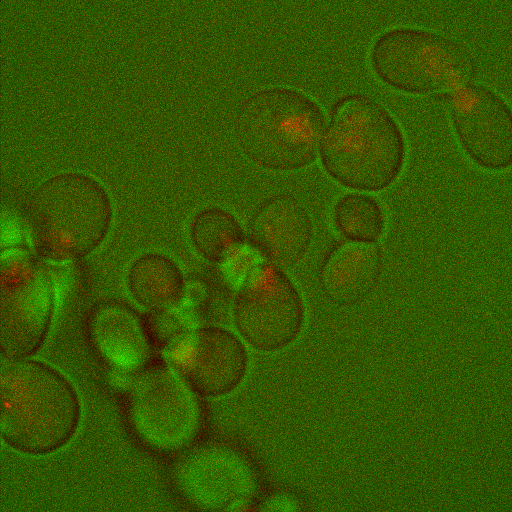

Supplement: Supplementary file 8 — Source data Fig. 3 [file 44318_2024_139_MOESM8_ESM.zip › Fig 3 data/Fig 3G_data/replicate 2/rad9del+sgs1/5.tif]

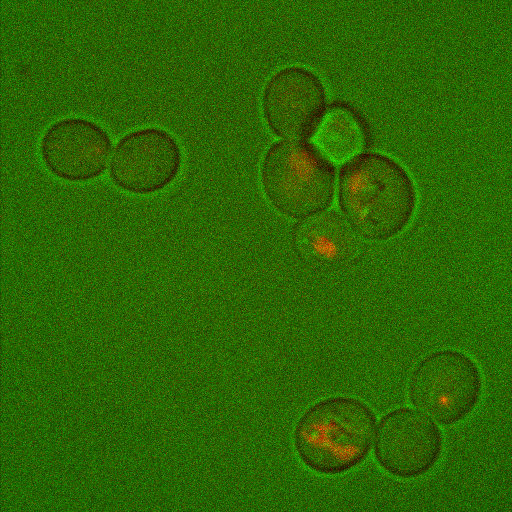

Supplement: Supplementary file 8 — Source data Fig. 3 [file 44318_2024_139_MOESM8_ESM.zip › Fig 3 data/Fig 3G_data/replicate 2/rad9del+sgs1/4.tif]

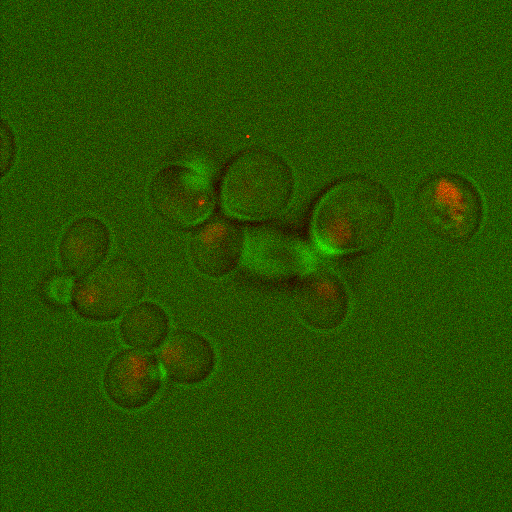

Supplement: Supplementary file 8 — Source data Fig. 3 [file 44318_2024_139_MOESM8_ESM.zip › Fig 3 data/Fig 3G_data/replicate 2/rad9del+sgs1/6.tif]

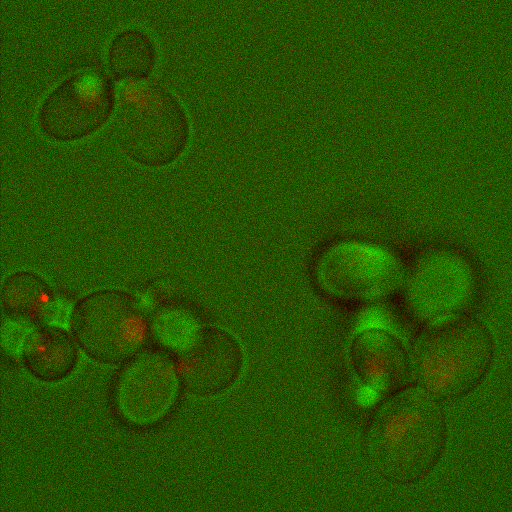

Supplement: Supplementary file 8 — Source data Fig. 3 [file 44318_2024_139_MOESM8_ESM.zip › Fig 3 data/Fig 3G_data/replicate 2/rad9del+sgs1/7.tif]

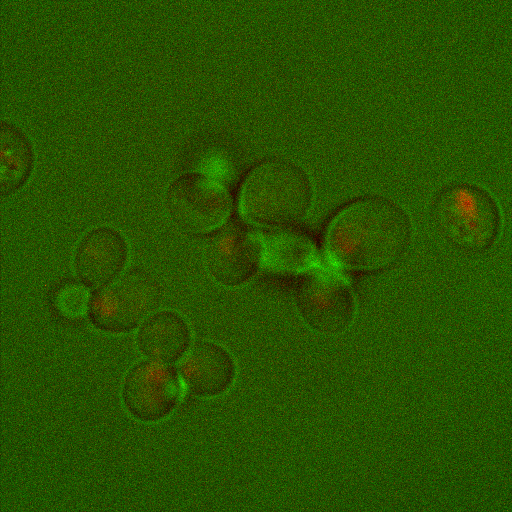

Supplement: Supplementary file 8 — Source data Fig. 3 [file 44318_2024_139_MOESM8_ESM.zip › Fig 3 data/Fig 3G_data/replicate 2/rad9del+sgs1/19.tif]

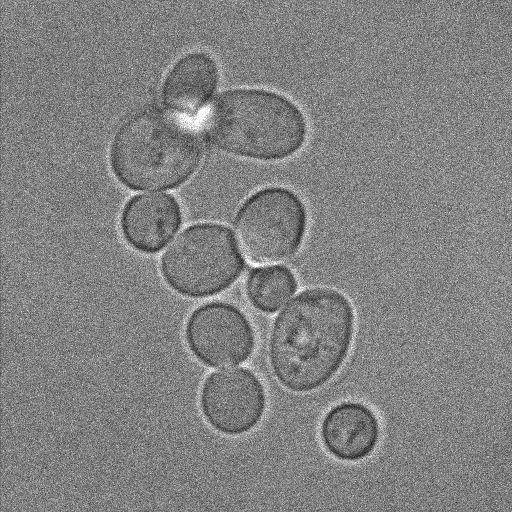

Supplement: Supplementary file 8 — Source data Fig. 3 [file 44318_2024_139_MOESM8_ESM.zip › Fig 3 data/Fig 3G_data/replicate 2/rad9del+sgs1/rep_image_Rad52_DIC.tif]

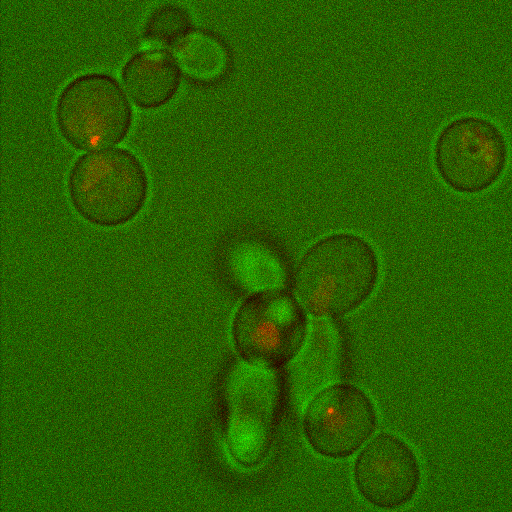

Supplement: Supplementary file 8 — Source data Fig. 3 [file 44318_2024_139_MOESM8_ESM.zip › Fig 3 data/Fig 3G_data/replicate 2/rad9del+sgs1/18.tif]

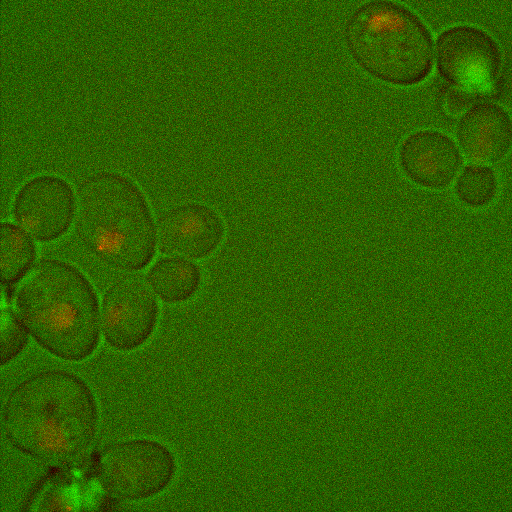

Supplement: Supplementary file 8 — Source data Fig. 3 [file 44318_2024_139_MOESM8_ESM.zip › Fig 3 data/Fig 3G_data/replicate 2/rad9del+sgs1/20.tif]

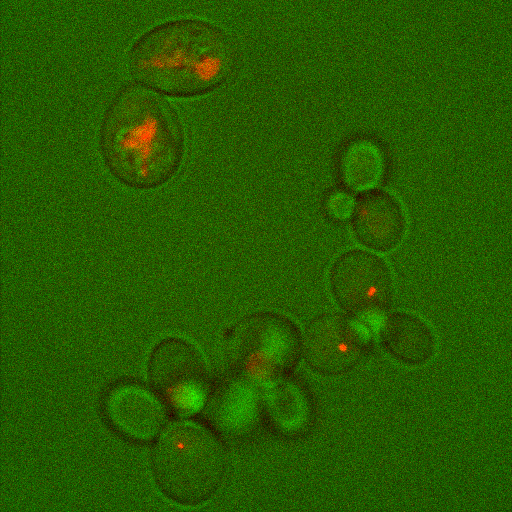

Supplement: Supplementary file 8 — Source data Fig. 3 [file 44318_2024_139_MOESM8_ESM.zip › Fig 3 data/Fig 3G_data/replicate 2/rad9del+D2-sgs1/13.tif]

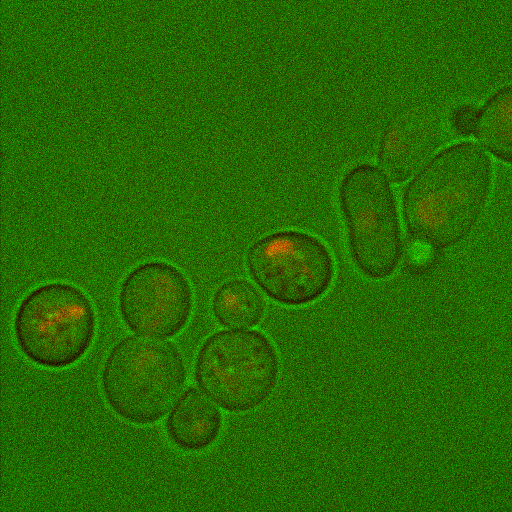

Supplement: Supplementary file 8 — Source data Fig. 3 [file 44318_2024_139_MOESM8_ESM.zip › Fig 3 data/Fig 3G_data/replicate 2/rad9del+D2-sgs1/12.tif]

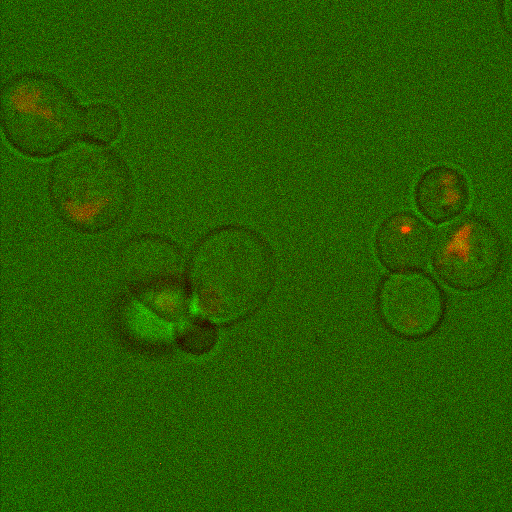

Supplement: Supplementary file 8 — Source data Fig. 3 [file 44318_2024_139_MOESM8_ESM.zip › Fig 3 data/Fig 3G_data/replicate 2/rad9del+D2-sgs1/10.tif]

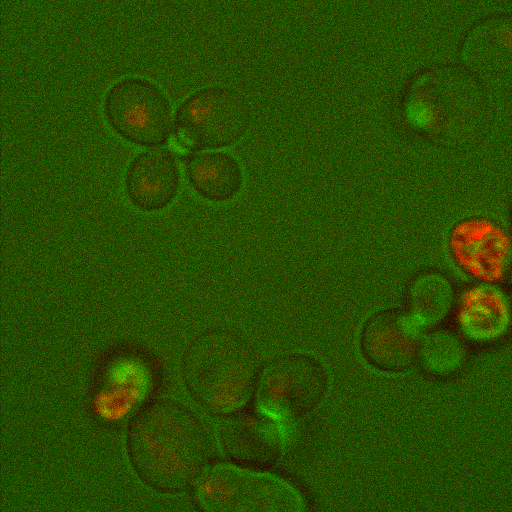

Supplement: Supplementary file 8 — Source data Fig. 3 [file 44318_2024_139_MOESM8_ESM.zip › Fig 3 data/Fig 3G_data/replicate 2/rad9del+D2-sgs1/11.tif]

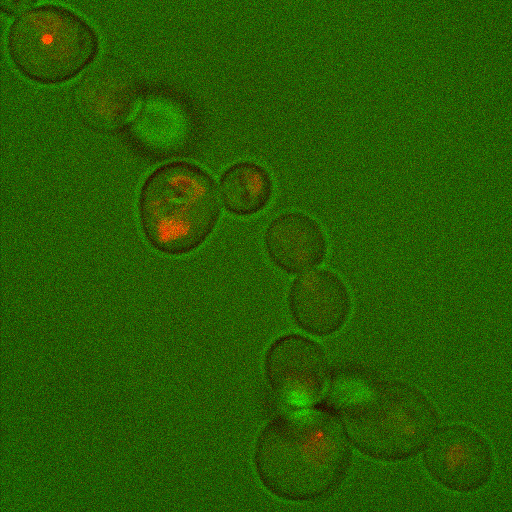

Supplement: Supplementary file 8 — Source data Fig. 3 [file 44318_2024_139_MOESM8_ESM.zip › Fig 3 data/Fig 3G_data/replicate 2/rad9del+D2-sgs1/15.tif]

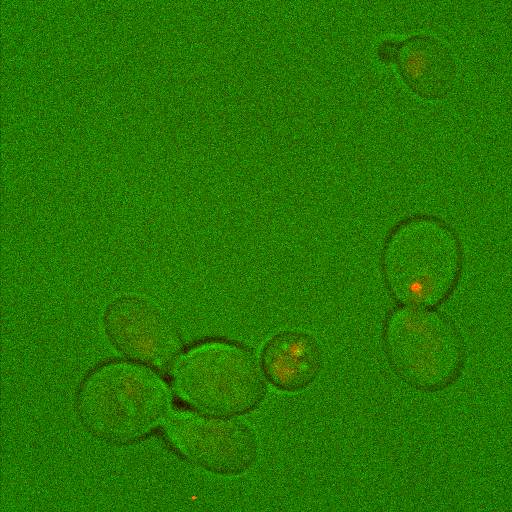

Supplement: Supplementary file 8 — Source data Fig. 3 [file 44318_2024_139_MOESM8_ESM.zip › Fig 3 data/Fig 3G_data/replicate 2/rad9del+D2-sgs1/14.tif]

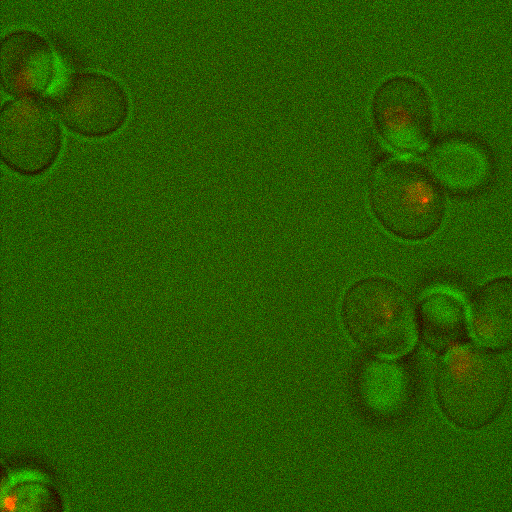

Supplement: Supplementary file 8 — Source data Fig. 3 [file 44318_2024_139_MOESM8_ESM.zip › Fig 3 data/Fig 3G_data/replicate 2/rad9del+D2-sgs1/16.tif]

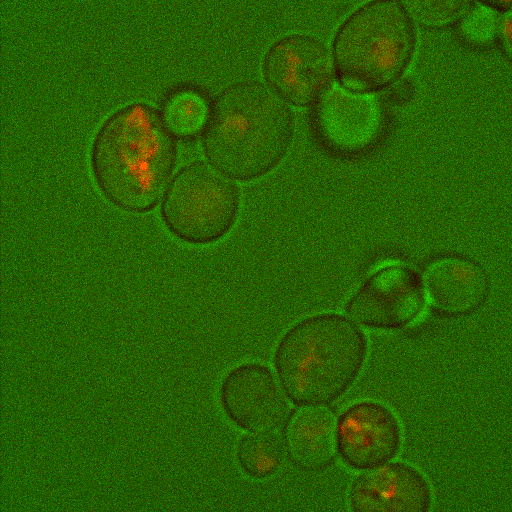

Supplement: Supplementary file 8 — Source data Fig. 3 [file 44318_2024_139_MOESM8_ESM.zip › Fig 3 data/Fig 3G_data/replicate 2/rad9del+D2-sgs1/17.tif]

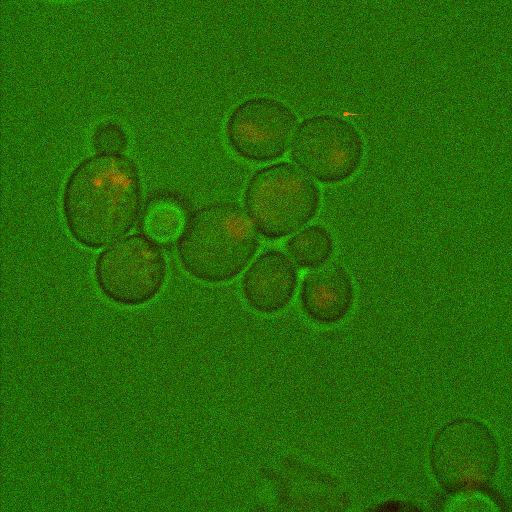

Supplement: Supplementary file 8 — Source data Fig. 3 [file 44318_2024_139_MOESM8_ESM.zip › Fig 3 data/Fig 3G_data/replicate 2/rad9del+D2-sgs1/9.tif]

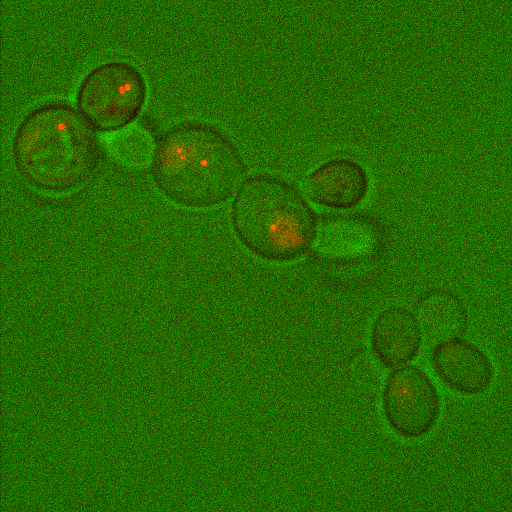

Supplement: Supplementary file 8 — Source data Fig. 3 [file 44318_2024_139_MOESM8_ESM.zip › Fig 3 data/Fig 3G_data/replicate 2/rad9del+D2-sgs1/8.tif]

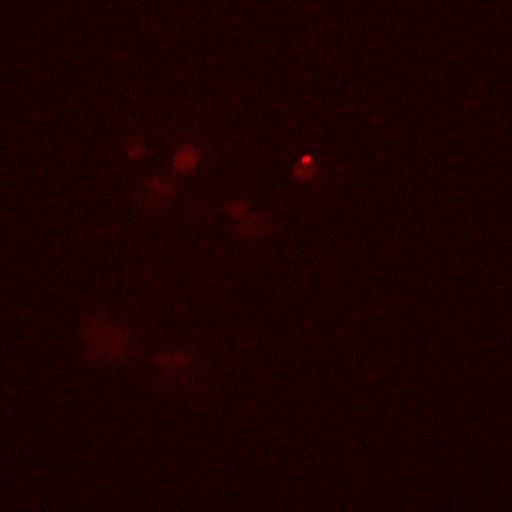

Supplement: Supplementary file 8 — Source data Fig. 3 [file 44318_2024_139_MOESM8_ESM.zip › Fig 3 data/Fig 3G_data/replicate 2/rad9del+D2-sgs1/rep_image_Rad52.tif]

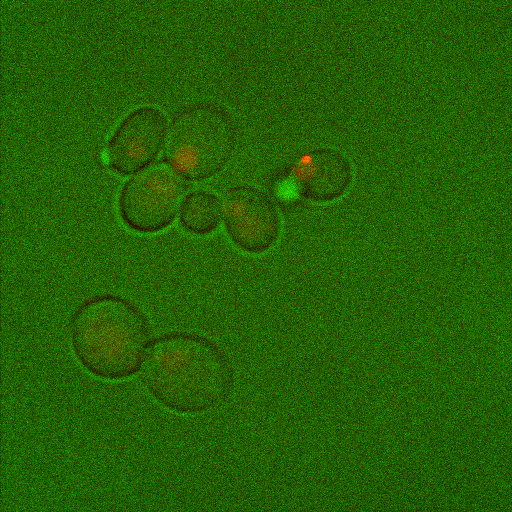

Supplement: Supplementary file 8 — Source data Fig. 3 [file 44318_2024_139_MOESM8_ESM.zip › Fig 3 data/Fig 3G_data/replicate 2/rad9del+D2-sgs1/3.tif]

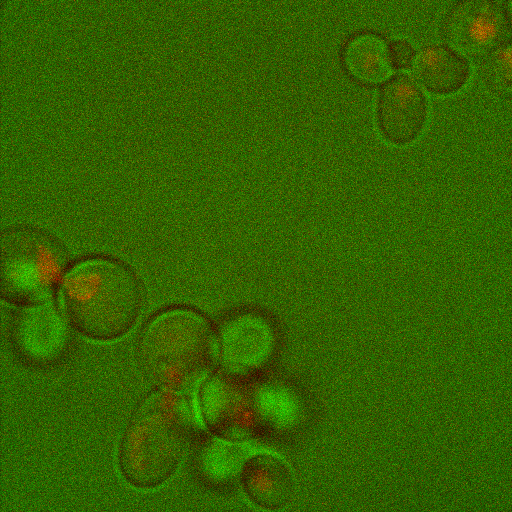

Supplement: Supplementary file 8 — Source data Fig. 3 [file 44318_2024_139_MOESM8_ESM.zip › Fig 3 data/Fig 3G_data/replicate 2/rad9del+D2-sgs1/2.tif]

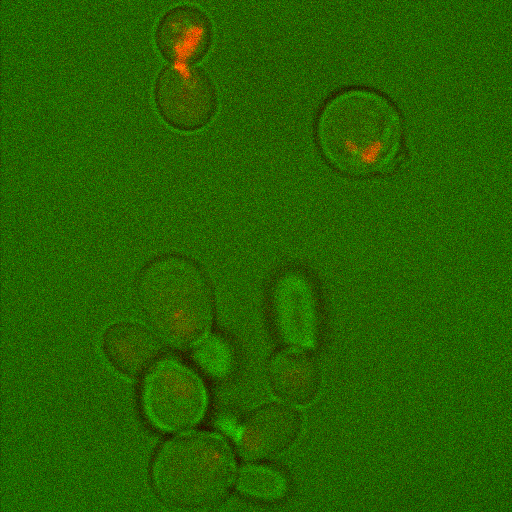

Supplement: Supplementary file 8 — Source data Fig. 3 [file 44318_2024_139_MOESM8_ESM.zip › Fig 3 data/Fig 3G_data/replicate 2/rad9del+D2-sgs1/1.tif]

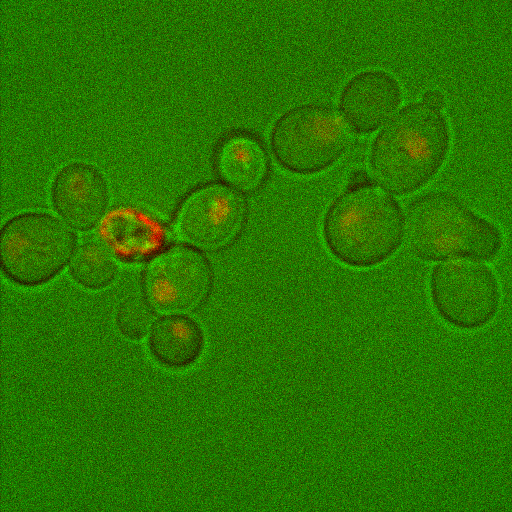

Supplement: Supplementary file 8 — Source data Fig. 3 [file 44318_2024_139_MOESM8_ESM.zip › Fig 3 data/Fig 3G_data/replicate 2/rad9del+D2-sgs1/5.tif]

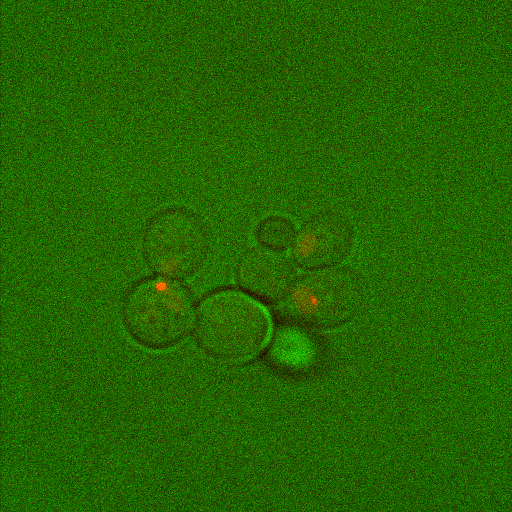

Supplement: Supplementary file 8 — Source data Fig. 3 [file 44318_2024_139_MOESM8_ESM.zip › Fig 3 data/Fig 3G_data/replicate 2/rad9del+D2-sgs1/4.tif]

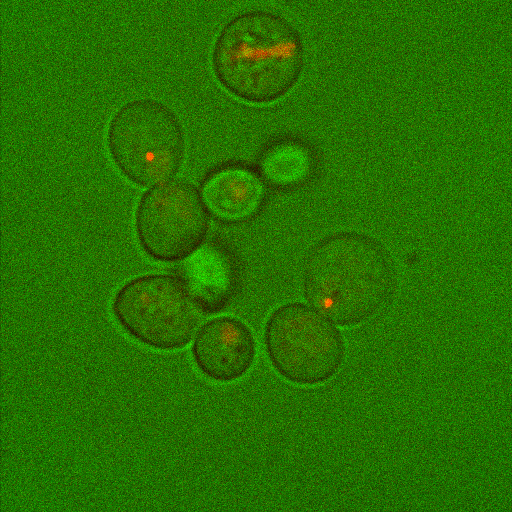

Supplement: Supplementary file 8 — Source data Fig. 3 [file 44318_2024_139_MOESM8_ESM.zip › Fig 3 data/Fig 3G_data/replicate 2/rad9del+D2-sgs1/6.tif]

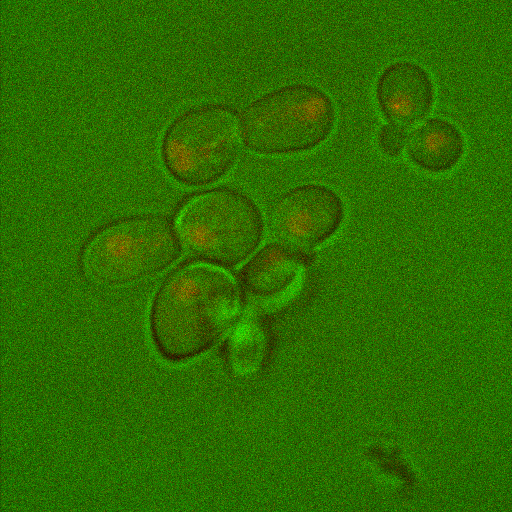

Supplement: Supplementary file 8 — Source data Fig. 3 [file 44318_2024_139_MOESM8_ESM.zip › Fig 3 data/Fig 3G_data/replicate 2/rad9del+D2-sgs1/7.tif]

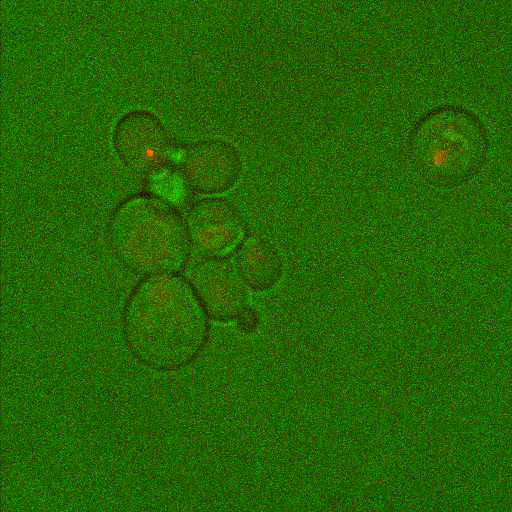

Supplement: Supplementary file 8 — Source data Fig. 3 [file 44318_2024_139_MOESM8_ESM.zip › Fig 3 data/Fig 3G_data/replicate 2/rad9del+D2-sgs1/19.tif]

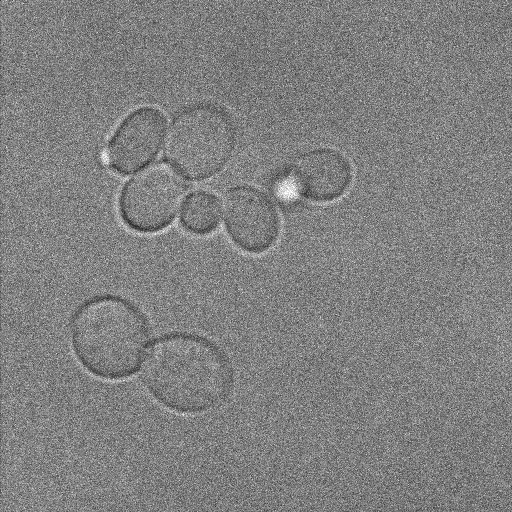

Supplement: Supplementary file 8 — Source data Fig. 3 [file 44318_2024_139_MOESM8_ESM.zip › Fig 3 data/Fig 3G_data/replicate 2/rad9del+D2-sgs1/rep_image_Rad52_DIC.tif]

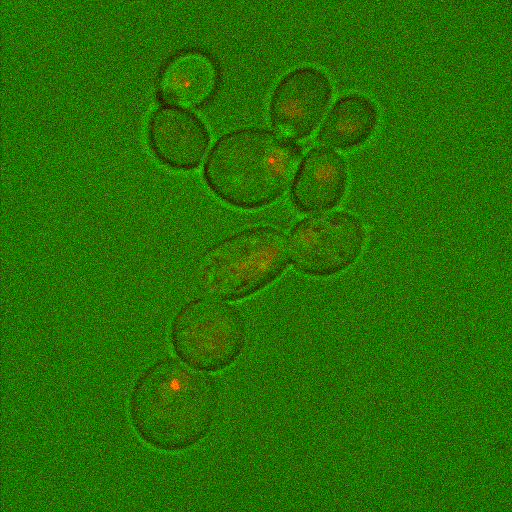

Supplement: Supplementary file 8 — Source data Fig. 3 [file 44318_2024_139_MOESM8_ESM.zip › Fig 3 data/Fig 3G_data/replicate 2/rad9del+D2-sgs1/18.tif]

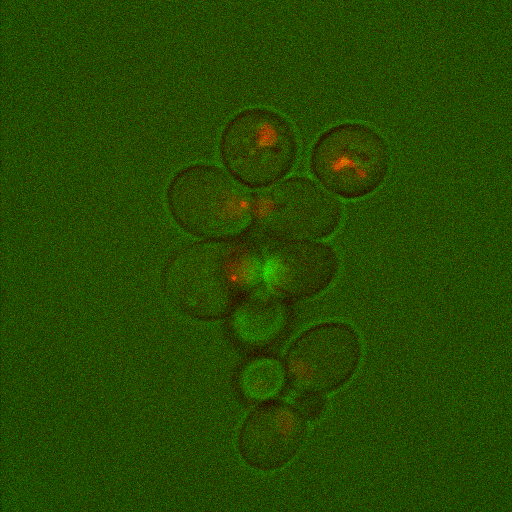

Supplement: Supplementary file 8 — Source data Fig. 3 [file 44318_2024_139_MOESM8_ESM.zip › Fig 3 data/Fig 3G_data/replicate 2/rad9del+D2-sgs1/20.tif]

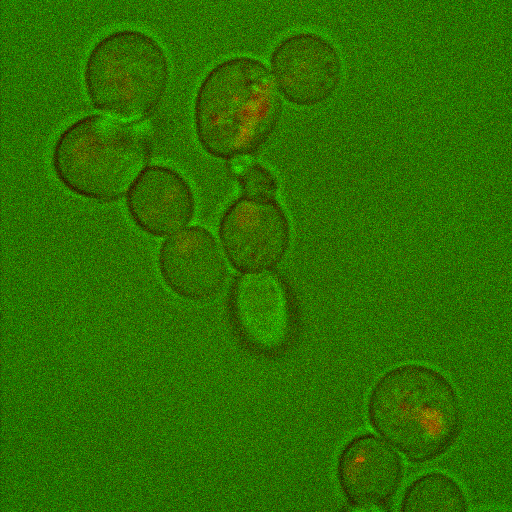

Supplement: Supplementary file 8 — Source data Fig. 3 [file 44318_2024_139_MOESM8_ESM.zip › Fig 3 data/Fig 3G_data/replicate 3/rad9del+sgs1+001MMS/13.tif]

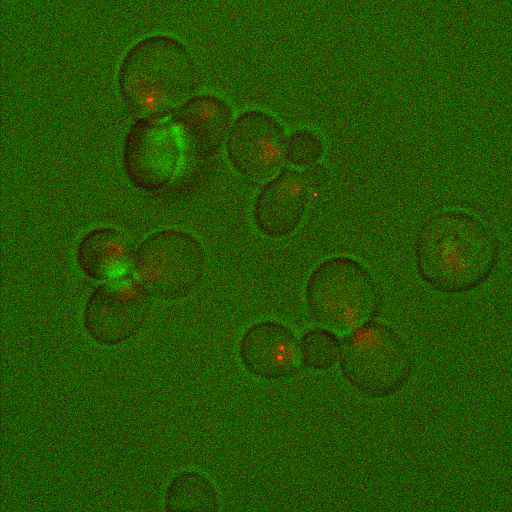

Supplement: Supplementary file 8 — Source data Fig. 3 [file 44318_2024_139_MOESM8_ESM.zip › Fig 3 data/Fig 3G_data/replicate 3/rad9del+sgs1+001MMS/12.tif]

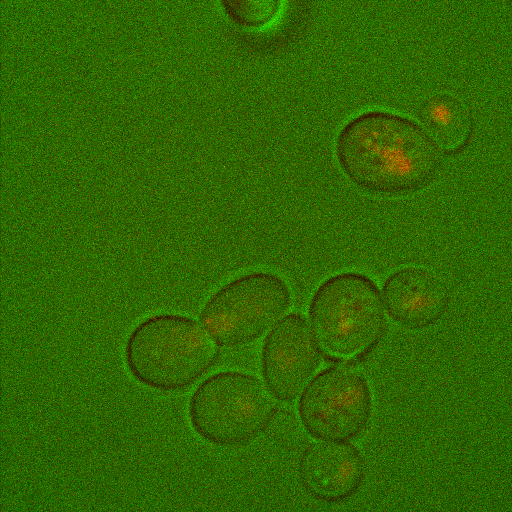

Supplement: Supplementary file 8 — Source data Fig. 3 [file 44318_2024_139_MOESM8_ESM.zip › Fig 3 data/Fig 3G_data/replicate 3/rad9del+sgs1+001MMS/10.tif]
